# Discovery of Novel Tyrosinase Inhibitors From Marine Cyanobacteria

**DOI:** 10.3389/fmicb.2022.912621

**Published:** 2022-07-13

**Authors:** Yifan He, Takashi L. Suyama, Hyunwoo Kim, Evgenia Glukhov, William H. Gerwick

**Affiliations:** ^1^Scripps Institution of Oceanography, University of California San Diego, La Jolla, CA, United States; ^2^Department of Chemistry and Forensic Science, Waynesburg University, Waynesburg, PA, United States; ^3^College of Pharmacy, Dongguk University, Goyang, South Korea; ^4^Skaggs School of Pharmacy and Pharmaceutical Sciences, University of California, San Diego, La Jolla, CA, United States

**Keywords:** marine cyanobacteria and algae, mushroom tyrosinase inhibition, kinetic study, synergistic effect, molecular docking, scytonemin monomer synthesis, skin whitening, scytonemin

## Abstract

Tyrosinase, an important oxidase involved in the primary immune response in humans, can sometimes become problematic as it can catalyze undesirable oxidation reactions. Therefore, for decades there has been a strong pharmaceutical interest in the discovery of novel inhibitors of this enzyme. Recent studies have also indicated that tyrosinase inhibitors can potentially be used in the treatment of melanoma cancer. Over the years, many new tyrosinase inhibitors have been discovered from various natural sources; however, marine natural products (MNPs) have contributed only a small number of promising candidates. Therefore, in this study we focused on the discovery of new MNP tyrosinase inhibitors of marine cyanobacterial and algal origins. A colorimetric tyrosinase inhibitory assay was used to screen over 4,500 marine extracts against mushroom tyrosinase (*A. bisporus*). Our results revealed that scytonemin monomer (ScyM), a pure compound from our compound library and also the monomeric last-step precursor in the biosynthesis of the well-known cyanobacterial sunscreen pigment “scytonemin,” consistently showed the highest tyrosinase inhibitory score. Determination of the half maximal inhibitory concentration (IC_50_) further indicated that ScyM is more potent than the commonly used commercial inhibitor standard “kojic acid” (KA; IC_50_ of ScyM: 4.90 μM vs. IC_50_ of KA: 11.31 μM). After a scaled-up chemical synthesis of ScyM as well as its *O*-methyl analog (ScyM-OMe), we conducted a series of follow-up studies on their structures, inhibitory properties, and mode of inhibition. Our results supported ScyM as the second case ever of a novel tyrosinase inhibitory compound based on a marine cyanobacterial natural product. The excellent *in vitro* performance of ScyM makes it a promising candidate for applications such as a skin-whitening agent or an adjuvant therapy for melanoma cancer treatment.

## Introduction

The market for skin-whitening products, especially in Asian countries, such as China, India, and Japan, is experiencing unprecedented growth (Grand View Research, 2019). The fundamental mechanism of such skin-whitening or dark-spot treatment products, is to reduce the amount of melanin in the epidermis. Produced in the specialized melanocytes cells, melanin describes a group of natural pigments that determines our skin, eye, and hair colors by the quantity and distribution of its container organelle termed melanosomes (Pillaiyar et al., 2017; Qian et al., 2020). Under normal physiological conditions, melanin production (also known as “melanogenesis”) plays a crucial role in protecting humans from UV-induced skin damage caused by harmful amounts of UV light exposure (Brenner and Hearing, 2008). However, melanin can become problematic when it is over-produced or is unevenly distributed, leading to undesirable skin problems include hyperpigmentation (i.e., freckles, age spots; Pillaiyar et al., 2017) and melanoma (Brenner and Hearing, 2008). Therefore, it is desirable to regulate the melanogenesis pathway to avoid deleterious effects while maintaining normal function.

**Figure 1:**
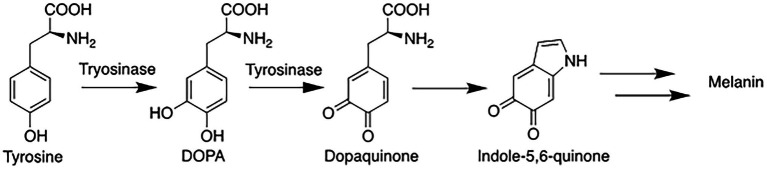
Simplified melanin biosynthesis pathway with tyrosinase catalyzing two of the early steps from the amino acid tyrosine.

A single enzyme known as tyrosinase is key to regulating the biosynthesis of melanin (Figure 1; Supplementary Figure S1). The melanogenesis pathway is initiated by the oxidation of the starting material l-tyrosine (or l-DOPA) to dopaquinone by tyrosinase (D'Mello et al., 2016); the resulting quinone then serves as the substrate for the subsequent steps that eventually leads to the production of melanin(s). Because the latter steps can proceed spontaneously at physiological pH once dopaquinone is produced (Pillaiyar et al., 2017), the formation of dopaquinone catalyzed by tyrosinase has been considered to be the rate-limiting step in melanin production.

Consequently, the search for tyrosinase inhibitors to restrain the activity of this enzyme and thereby restrict the production of melanin has been long considered a desired approach for treating hyperpigmentation (Zolghadri et al., 2019). However, even after being studied for a considerable time, tyrosinase inhibitors that are currently used in skin care products still have drawbacks. Patients with sensitive skin may develop dermatitis, allergy, and even more severe conditions such as cancer (Cho et al., 2020). Therefore, it remains an active topic in the pharmaceutical field to find more and better tyrosinase inhibitors. Over the years, a number of new tyrosinase inhibitors have been isolated from various natural sources, but only a few candidates come from marine natural products (MNPs; Shimoda et al., 2010; Deering et al., 2016). In this regard, the exploration of tyrosinase inhibitors from marine cyanobacteria has been especially largely understudied, with only one case of oscillapeptin G reported to date (Sano and Kaya, 1996).

**Figure 2:**
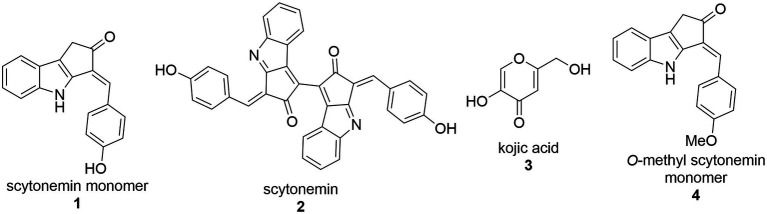
The structures of compounds **1**–**4**.

**Scheme 1:**
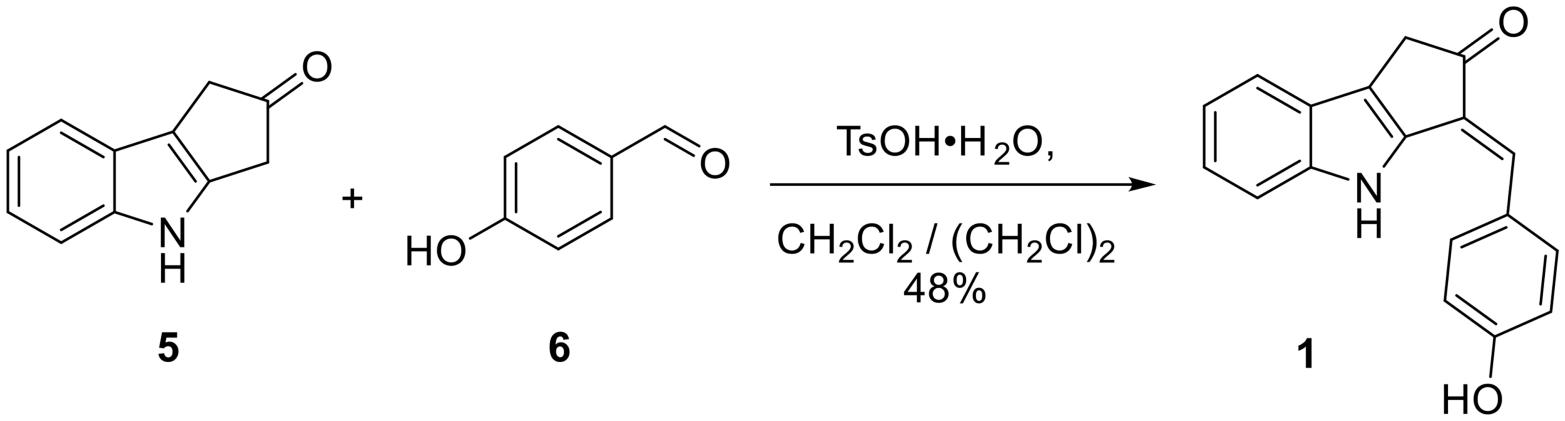
One-step synthesis of ScyM **1** from indole **5** and 4-hydroxybenzaldehyde **6**.

**Scheme 2:**
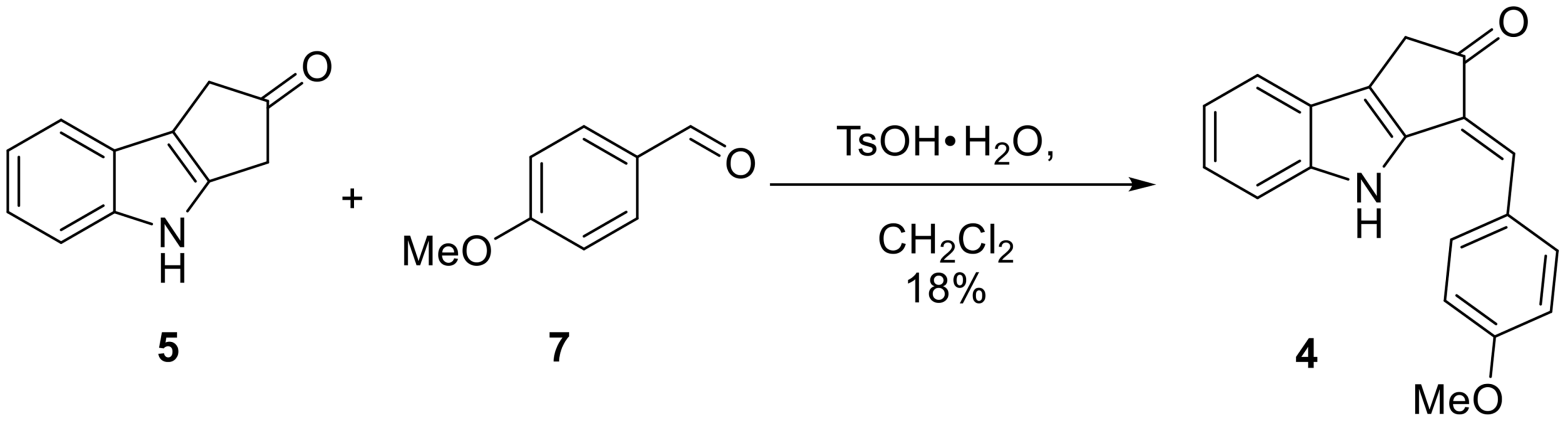
One-step synthesis of ScyM-OMe **4** from indole **5** and anisaldehyde **7**.

With the demand for more effective tyrosinase inhibitors and the under-explored nature of marine algae and cyanobacteria for compounds with this activity, we undertook a screen of over 4,500 marine extracts in our extract and pure compound library against mushroom tyrosinase (*A. bisporus*), using a well-validated classic colorimetric fungal-tyrosinase inhibitory assay. After multiple validations through dose-dependent testing, our results revealed that scytonemin monomer (ScyM, **1**; Figure 2), a pure compound from our library and the monomeric last-step precursor in the biosynthesis of the well-known cyanobacterial sunscreen pigment scytonemin (**2**; Figure 2), consistently showed the highest tyrosinase inhibitory score, and was even more potent than the commonly used commercial inhibitor standard kojic acid (KA, **3**; Figure 2). We then conducted a new scaled-up chemical synthesis of ScyM as well as its *O*-methylated analog ScyM-OMe (**4**; Figure 2), whose structures were confirmed *via* extensive 1D and 2D NMR and MS analysis. The synthesis of **1** and **4** reported here in Schemes 1 and 2, was achieved in one step from commercially available starting materials with satisfactory yields. Further biochemical assay work, in conjunction with *in silico* docking and an enzyme kinetics study, suggests that ScyM shows a slowly reversible mixed-type inhibition against mushroom tyrosinase, with its phenol moiety being indispensable for its excellent inhibitory effect. Thus, we successfully identified ScyM as the second case of a novel tyrosinase inhibitory compound based on a marine cyanobacterial natural product. This work opens the possibility that future research on other cyanobacterial-derived natural products will reveal additional anti-tyrosinase compounds for pharmaceutical and cosmeceutical applications.

## Materials and Methods

### General Experimental Procedures

All starting materials were purchased from Sigma-Aldrich. Other synthetic reagents and solvents were purchased from Fischer Scientific. All solvents employed in synthesis were dried by distilling from CaH_2_ immediately prior to use. LCMS analyses were carried out on a Thermo Finnigan Surveyor Autosampler-Plus/LC-Pump-Plus/PDA-Plus system and a Finnigan LCQ Advantage Plus mass spectrometer, with samples dissolved at 1 mg/ml in MeOH. Reversed-phase HPLC was performed on a Kinetex C18 semipreparative column (100 × 4.6 mm × 5 μm, Phenomenex) using a Thermo Fisher Scientific HPLC system comprising a Thermo Dionex UltiMate 3000 pump, RS autosampler, RS diode array detector, and automated fraction collector. 1D NMR and 2D NMR spectra were acquired using a Bruker Avance III DRX-600 NMR and a JEOL ECZ 500 NMR spectrometer. Deuterated solvents for NMR were purchased from Cambridge Isotope Laboratories, Inc. Samples were passed through C18 SPE (5,000 mg/20 ml, SEClute) before LC injections. All other general solvents were HPLC grade as purchased from Thermo Fisher Scientific.

### Cyanobacterial/Algal Materials and Reagents

Most of the compounds, fractions and extracts used in this Medium Throughput Anti-tyrosinase Screening campaign were purified from a variety of tropical marine cyanobacterial and algal collections produced in our laboratory during past expeditions. A few of the compounds from the collection of Pure Compounds had been synthesized by former researchers in our laboratory. All samples were stored in DMSO at −80°C before dilution and testing. Mushroom tyrosinase (polyphenol oxidase) was obtained from Sigma-Aldrich and Worthington Biochemical Corp. The substrate 3-(3,4-Dihydroxyphenyl)-l-alanine (l-DOPA) was obtained from Tokyo Chemical Industry Corp. Sodium Phosphate buffer (0.1 M, pH 6.8) was purchased from bioWORLD Corp. The positive control compound, Kojic Acid (KA), was purchased from Thermo Fisher Scientific Corporation. DMSO from Sigma-Aldrich was used in small amounts to dissolve samples as well as in controls for consistency within the assay procedure. A Millipore Milli-Q system (Burlington, MA, United States) was used to purify water for use in dilutions.

### Synthesis of Scytonemin Monomer (**1**)

To a solution of the indole **5** (57 mg, 0.333 mmol, 1 eq) in 12 ml of 4:1 CH_2_CH_2_/ (CH_2_Cl)_2_ in a round bottom flask under N_2_ at RT were added 4-hydroxybenzaldehyde (**6**, 45 mg, 0.368 mmol, 1.1 eq) and TsO·H_2_O (7.8 mg, 0.041 mmol, 12 mol%). The flask was then equipped with a Hickman still holding 3 Å molecular sieves and a condenser on top of the Hickman still. The reaction mixture was then refluxed under vigorous stirring overnight. Upon cooling to RT, the reaction mixture was filtered through a plug of silica gel and rinsed with EtOAc. The volatiles were removed under vacuum from the combined crude product solution. The crude solid was dissolved in 1:9 MeOH/CH_2_Cl_2_ and subjected to silica gel flash column chromatography employing a gradient mobile phase from 100% hexanes to 1:1:18 EtOAc/CH_2_Cl_2_/hexanes, then to 1:10:10:80 MeOH/EtOAc/CH_2_Cl_2_/hexanes, and then finally to 1:3:3:6 MeOH/EtOAc/CH_2_Cl_2_/hexanes. A yellow-brownish solid (44 mg, 0.160 mmol, 48% yield) was obtained. This material contained a trace amount of starting material **6** and was therefore further purified by reverse-phased HPLC with an isocratic mobile phase consisting of 1:1 acetonitrile/H_2_O containing 0.1% formic acid. Pure synthetic compound **1** showed the following: TLC R_f_ = 0.54 (1:1 EtOAc/hexanes); Mp > 210°C; IR (film, KBr) ν_max_ 3,375 (br), 2,963, 2,921, 1712, 1,599, 1,483, 1,441, 1,274, 1,231, 1,165, 1,123, 745 cm^−1^; ^1^H and ^13^C NMR data, see Table 1.

**Table 1:** Observed and expected (as reported in Suyama, 2009) ^1^H and ^13^C NMR spectroscopic data for scytonemin monomer (ScyM, **1**) in DMF-d_7_.

Carbon position	Observed in this study		Expected (Suyama, 2009)	
	*δ*_C_, type	*δ*_H_ (*J* in Hz)	*δ*_C_, type	*δ*_H_ (*J* in Hz)
1	141.44, C		141.6, C	
2	141.25, C		141.4, C	
3	127.07, C		127.3, C	
4	205.26, C		205.3, C	
5	36.76, CH_2_	3.56, s	36.9, CH_2_	3.56, s
6	120.64, C		120.9, C	
7	125.03, C		125.2, C	
8	120.20, CH	7.57, d (7.89)	120.4, CH	7.57, d (8.0)
9	121.08, CH	7.12, ddd (7.54, 7.21, 1.02)	121.2, CH	7.12, ddd (7.9, 4.5, 0.6)
10	124.34, CH	7.23, ddd (8.25, 6.91, 1.22)	124.4, CH	7.23, ddd (8.2, 7.2, 1.3)
11	113.82, CH	7.61, d (8.28)	113.9, CH	7.60, d (8.1)
12	125.36, CH	7.06, s	125.4, CH	7.06, s
13	127.26, C		127.6, C	
14, 18	131.58, CH	7.70, d (8.66)	131.7, CH	7.70, d (8.5)
15, 17	117.42, CH	6.98, d (8.55)	117.3, CH	6.97, dd (6.1, 2.4)
16	160.31, C		160.2, C	

### Synthesis of *O*-Methyl Scytonemin Monomer (**4**)

To a solution of the indole **5** (52 mg, 0.304 mmol) in CH_2_Cl_2_ (10 ml) under N_2_ in a round bottom flask at RT were added anisaldehyde (**7**, 40 μl, 0.330 mmol, 1.1 eq) and TsO·H_2_O (6 mg, 0.0315 mmol, 10 mol%). This flask was then equipped with a Hickman still holding 3 Å molecular sieves and a condenser on top of the Hickman still. The reaction mixture was refluxed overnight. Most of the solvent had evaporated at this point, leaving behind a dark brown solid. The crude material was dissolved in CH_2_Cl_2_ and subjected to silica gel flash column chromatography with a gradient mobile phase ranging from 1:49 acetone/hexanes to 1:4 acetone/hexanes. A yellow solid (16 mg, 0.0553 mmol, 18% yield) was obtained that showed the following: TLC R_f_ = 0.40 (1:3 acetone/hexanes); ^1^H NMR (300 MHz, CDCl_3_) δ 8.21 (s, 1H), 7.60 (d, *J* = 8.8 Hz, 2H), 7.55 (d, *J* = 8.0 Hz, 1H), 7.35 (d, *J* = 8.1 Hz, 1H), 7.25 (t, *J* = 7.2 Hz, 1H), 7.17 (s, 1H), 7.16 (dd, *J* = 7.4 Hz, 1H), 7.04 (d, *J* = 8.7 Hz, 2H), 3.90 (s, 3H), 3.55 (s, 2H); ^13^C NMR (75 MHz, CDCl_3_) δ 204.6, 160.3, 140.2, 138.8, 129.6, 128.7, 127.8, 124.3, 124.3, 120.9, 120.3, 119.8, 114.8, 111.8, 55.5, 36.5; HRMS (EI) calcd for C_19_H_15_NO_2_Na [M + Na]^+^ 312.0995, found 312.0998 (Δ1.0 ppm).

### End-Point Colorimetric Tyrosinase Inhibition Assay for Broad Screening Campaign

Tyrosinase inhibitory activity of the fractions or pure compounds was evaluated using l-DOPA as substrate. Experiments were constructed in 96-well microplates format with slight modifications made from previously described methods (Masuda et al., 2005; Mutschlechner et al., 2018). Four types of assay wells were designated for each screening plate (see Supplementary Figure S2 for visualization), in which the following reaction mixtures were added: A [Blank Control with Enzyme] contains 2 μl of DMSO, 118 μl of prepared 0.067 M sodium phosphate buffer and 40 μl of tyrosinase (100 units/ml) in the same buffer; B [Blank Control without Enzyme] contains 2 μl of DMSO and 158 μl of 0.067 M sodium phosphate buffer; C [Testing Material with Enzyme] contains 2 μl of sample (1 mg/ml in DMSO), 118 μl of 0.067 M sodium phosphate buffer and 40 μl of tyrosinase (100 Units/mL) in the same buffer; D [Testing Material without Enzyme] contains 2 μl of sample (1 mg/ml in DMSO) and 158 μl of 0.067 M sodium phosphate buffer. Samples were tested in triplicate.

The contents of each well were first mixed and pre-incubated at RT for 20 min after which 40 μl of 3 mM l-DOPA was added as substrate to the reaction mixture, bringing the volume of each well to 200 μl. The final test sample concentration was 10 μg/ml, while the final DMSO concentrations for both negative controls and samples kept ≤1%. Twenty minutes after addition of the l-DOPA, the plate was read on a SpectraMax M3 plate reader (Molecular Devices, San Jose, CA, United States) for absorbance measurement of each well at 475 nm (for actual calculation) and 490 nm (as a wavelength duplicate) in the end-point mode. Forty-five seconds of shaking was performed by the instrument before the wells were read.

The percentage inhibition of the tyrosinase activity was calculated by the equation: % Inhibition = {[(A − B) − (C − D)]/(A − B)} × 100, where A, B, C and D are all OD values from the corresponding wells at the same time point. It reflects the inhibitory strength of the testing material by comparing to the blank condition where no inhibitor is added.

### Dynamic Dose-Dependent Tyrosinase Inhibition Study

Similar to the end-point assays, the dynamic tyrosinase inhibitory activity of materials was also evaluated using l-DOPA as substrate. Experiments were carried in triplicate in 96-well microplates using the same 200 μl reagent mixture system as described in the broad screening section, with the difference being that the concentration of the sample (test material or positive control KA) was varied within the 2 μl of sample that was added to each well. Respectively, the final concentration gradient of the test samples was set to be 200, 150, 75, 25, 5, and 1 μM. For OD measurements, the plate was read in the dynamic mode for 60 min with measurements recorded every minute, using the SpectraMax M3 plate reader at 475 nm and 490 nm. Forty-five seconds of shaking was also performed before first reading to retain method consistency. The IC_50_ values and curves of average percent inhibition (triplicates) versus sample concentration were generated using data obtained at either 5 min or 20 min with GraphPad Prism 9.2.0.

### Kinetic Analysis of Tyrosinase and Inhibitors

The mode of inhibition and inhibition parameters [Michaelis–Menten constant (*K*_m_) and maximal velocity (V_max_)] of tyrosinase were determined by Lineweaver–Burk plot analysis using various concentrations of l-DOPA (0.15, 0.3 and 0.6 mM) as substrate. To accommodate for solubility issues, the inhibitor concentrations for ScyM were 10, 5, 2.5, 1.25 and 0 μM; for KA the concentrations were 25, 10, 5, 2.5 and 0 μM. The Lineweaver–Burk plot was generated using Graphpad Prism 9.2.0. Experiments were performed in triplicate.

### Molecular Docking Experiments

All docking analyses were carried out using the Molecular Operating Environment 2019 (MOE 2019.0102) program in which the docking results were manipulated using the GBVI/WSA dG scoring function that estimates the free binding energy of the ligand from a given orientation. Among the various tyrosinase enzyme crystal structures available in the Protein Data Bank, the *A. bisporus* tyrosinase enzyme co-crystalized with built-in tropolone inhibitors (PDB code: 2Y9X) was selected for molecular docking studies. This was consistent with the tyrosinase inhibition studies which all used the same mushroom tyrosinase. 3D structures of l-DOPA, KA, scytonemin, ScyM, and ScyM-OMe were prepared using the Builder module with energy minimization. Docking was performed with 2,000 poses once ligands were securely positioned in the enzyme binding pocket, and the best five poses were decided based on the negative binding free energy value (S value) and then selected to analyze receptor–ligand interactions. The cognate ligand inside 2Y9X, tropolone, was re-docked in the pocket as a validation of docking accuracy. Accordingly, a reasonable S value of −5.95 was obtained for the re-docked tropolone, and the resulted binding poses along with ligand interactions were close to its original reference conformation [root-mean-square deviation (RMSD) = 1.02 Å] (Ismaya et al., 2011).

### Cytotoxicity Assay

The cytotoxicity assay was performed using a protocol previously published (Yu H.-B. et al., 2019; Leber et al., 2020). Briefly, the MTT staining method was used to determine the cytotoxicity of compounds of interest using the NCI-H460 human lung carcinoma cell line purchased from ATCC (Manassas, VA, United States). The cells were grown in flasks and then seeded into wells at 3.33 × 10^4^ cells/ml of Roswell Park Memorial Institute (RPMI-1640) medium with standard fetal bovine serum (FBS), leading to 180 μl/well. The cells were then pre-incubated for 24 h at 37°C in 96-well plates before drug was added. Thereafter, with addition of diluted compounds of interest dissolved in DMSO, plates were incubated for an additional 48 h and then stained with MTT (thiazolyl blue tetrazolium bromide 98%; Sigma-Aldrich) for 25 min, after which the optical densities (OD) were recorded at 630 and 570 nm for each well on a SpectraMax M3 plate reader (Molecular Devices, San Jose, CA, United States). Doxorubicin and 1% DMSO in RPMI-1640 without fetal bovine serum were used as positive and negative controls, respectively. Polyethylene glycol (PEG) was used to facilitate dissolution of compounds with limited solubility.

### Statistical Analysis

Tyrosinase inhibitory scores of the best candidates from the broad screening were analyzed using ordinary one-way analysis of variance (ANOVA) on Graphpad Prism 9.2.0. The Tukey–Kramer multiple comparison post-hoc test was used to determine the significant difference among groups. *p* < 0.05 were considered to be statistically significant.

## Results and Discussion

### Background on the UV-Sunscreen Pigment Scytonemin **(2)**

Scytonemin, the pigment material found in the sheaths of many species of cyanobacteria, is a potent UV-absorbing metabolite that is used as a defense against UV radiation, and is often referred to as a “cyanobacterial sunscreen pigment” due to this property (Proteau et al., 1993). Additionally, it is also considered as a biogeochemical marker as a result of its stable and ancient nature, as well as its “recalcitrancy to degradation” (Garcia-Pichel and Castenholz, 1991). Although its existence was discovered early in the 19th century (Nägeli, 1849), because of its complex structure, the dimeric indole phenolic skeleton of scytonemin was not elucidated until 1993 by our group. This newly found skeletal type is composed of both indolic and phenolic subunits and was named the “scytoneman skeleton” (Proteau et al., 1993).

Motivated by its unique and complex structure, study of the biosynthesis of scytonemin was of great interest and was conducted at both the genomic and mechanistic level in several research laboratories (Balskus and Walsh, 2008; Sorrels et al., 2009; Soule et al., 2009b; Supplementary Figure S3). An upstream two-component regulatory system (Npun_F1277 and Npun_F1278) was discovered and shown to regulate the expression of the main 18-gene scytonemin cluster, while another set of five satellite genes (Npun_F5232 to Npun_F5236) was also considered to be involved in scytonemin biosynthesis (Soule et al., 2009a; Janssen and Soule, 2016). These satellite genes were referred as *ebo* genes in a recent study, in which the *ebo* genes were proven to be responsible for the export of the final precursor from the cytoplasm to the periplasm, the location where the final steps of scytonemin biosynthesis takes place (Klicki et al., 2018). Relative to this present study, the final step in this pathway was thought to form scytonemin through oxidative dimerization of monomeric species. However, a clear mechanism for this last dimerization step has not yet been determined, especially given that subjection of ScyM to various oxidizing conditions failed to yield scytonemin (Suyama, 2009). Moreover, the specific enzyme responsible for catalyzing the coupling of two monomers is still missing. While ScyE was proven to be crucial for scytonemin biosynthesis, its exact role remains ambiguous; ScyD and ScyF were reported to be non-essential for the last steps of scytonemin biosynthesis and might be redundant genes (Ferreira and Garcia-Pichel, 2016).

The biological activities of scytonemin have been reported in a number of reports since its discovery and characterization. It has been shown to possess anti-inflammatory, anti-tumor and calcium antagonistic properties by different research laboratories (Helms et al., 1988; Stevenson et al., 2002; Itoh et al., 2014), in addition to its UV-sunscreen properties (Garcia-Pichel and Castenholz, 1991). However, there has been limited biological testing of ScyM, the proposed final precursor in the scytonemin biosynthetic pathway. This might be because the chemical synthesis and sustainable production of scytonemin monomer was only recently published such that access to this material has been fairly restricted (Malla and Sommer, 2014; Ekebergh et al., 2015). Our synthetic approach took advantage of regioselective enol formation from **5**, resulting in a single regioisomer as the aldol condensation product (**1**) as seen in Scheme 1, and likewise for the *O*-methyl analog **4** in Scheme 2. This allowed us to drastically reduce the number of requisite steps to produce this important compound from 7 to 8 steps (Ekebergh et al., 2011, 2012, 2015) to a single step, paving the way for this current study. With adequate amounts of the compounds in hand, as well as the promising tyrosinase inhibitory score of ScyM from the initial screening, we further investigated the tyrosinase inhibitory activity of ScyM. These investigations included: (1) confirming its dose-dependent response and generating a reliable IC_50_ value for comparison with other inhibitors, (2) using Lineweaver-Burk plots for kinetic analysis on its mode of inhibition, and (3) deducing its potential positioning inside the tyrosinase binding site through *in silico* molecular docking.

### Assay Validation With KA **(3)**

Before the formal screening of our samples, several test trials were performed using the positive control KA so as to validate the experimental design and evaluate the performance of the reagents. The validating experiments used the same experimental setup as for the dose-dependent assay experiments, with KA being tested at various concentrations (i.e., 20, 10, 5, 2.5 and 1 μg/ml). After raw data processing, the inhibitory activity of KA at various concentrations (see Supplementary Table S1) was compared to literature values from preceding studies (Neeley et al., 2009), with data points fit onto a logarithmic curve (Supplementary Figure S4; IC_50_ = 5.4 μg/ml = 37.9 μM). These experiments established that the protocol was working properly, and thus formal screening of natural product samples could proceed.

### Initial Broad Screening Campaign

**Figure 3:**
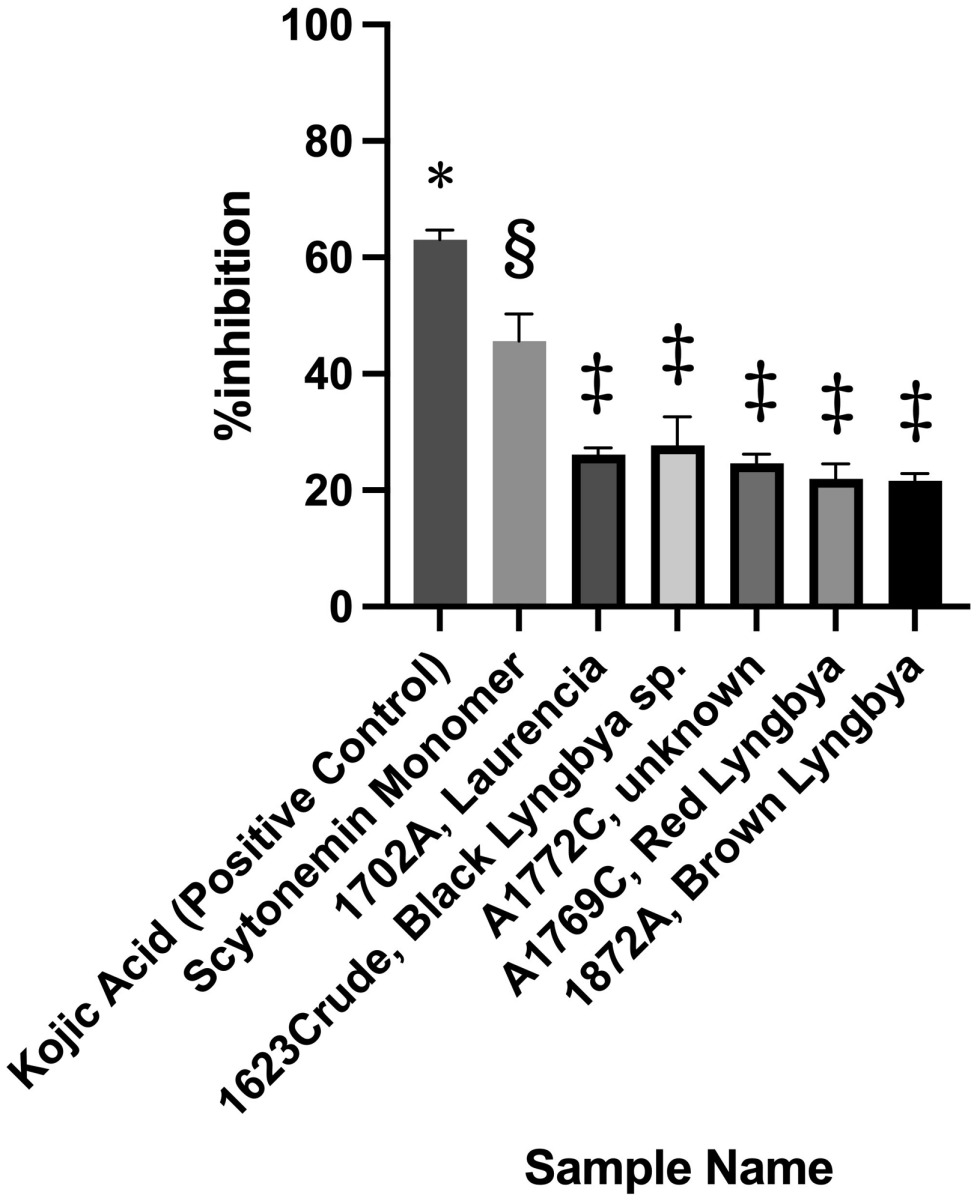
Tyrosinase inhibitory activity of the best candidates from the broad screening of extracts, fractions and pure compounds at 10 μg/ml. Error bars represent standard deviation. Symbols (*, §, ‡) indicate significant difference (at 0.05 level) by the Tukey–Kramer multiple comparison test. * vs. other groups, *p* < 0.001; § vs. other groups, *p* < 0.001. No significant difference is noted between groups labeled with same symbol.

For the broad screening campaign, over 4,500 extracts and fractions and all 169 pure compounds in the UCSD Marine Natural Product library were tested in the assay. Among all of these samples, only six passed our pre-set criterion of 20% inhibition, with scytonemin monomer (ScyM) being the only pure compound. By comparison, the KA control showed >60% inhibition at 10 μg/ml (see Figure 3). The five crude extracts or fractions, as illustrated in Figure 3, only barely passed the 20% inhibition threshold at 10 μg/ml and were later shown to be false positives after careful re-evaluations. On the other hand, ScyM consistently showed an inhibition level of ~45% at a screening dose of 10 μg/ml. Together with its statistical significance, ScyM thus stood out from all other primary hits and became our compound of primary interest.

Although multiple re-tests of ScyM all showed that it retained >20% inhibition, because ScyM is a colored material, there was concern that its pigment nature could interfere with the colorimetric assay. Therefore, the OD shifts for six different conditions over 20 min were monitored to prove ScyM’s promising inhibitory activity was a real inhibition and not the result of pigment absorption (see Supplementary Figure S5 and notes for details). Briefly, while its OD changes were evident when the tyrosinase enzyme was added, ScyM showed stable OD values when tyrosinase was not present (Negative Control). The result of this negative control experiment for ScyM was consistent with the result for KA, as well as for the blank control where no inhibitor was introduced (Supplementary Figures S5B,D,F), confirming that there were no OD fluctuations over time or significantly increased OD readings for ScyM. Therefore, we concluded that there was no pigment interference with the assay and the results were worthy of further analysis. It is also important to note that the final concentrations of samples and controls in this general screening effort were all at 10 μg/ml. Taking into account the molecular weight differences of ScyM (**1**) and KA (**3**) suggested that additional experiments were required to evaluate their relative potencies, and this required the chemical synthesis of additional ScyM (**1**) as detailed below.

### Chemical Synthesis of Scytonemin Monomer and Purification

In the positive mode ESI MS spectrum, ScyM (exact mass = 275.10) showed a parent peak at *m/z* 275, while the MS^2^ spectrum showed a detailed pattern and suggested a potential [M-28]^+^ fragmentation pattern (Supplementary Figures S6B S7). This base peak and MS^2^ pattern matched perfectly with previously recorded high resolution MS data when ScyM (**1**) was first produced, which was also performed in the positive ion ESI MS mode (Suyama, 2009). We hypothesize that the [M-28]^+^ peak at *m/z* 247 might derive from an unusual loss of CO that is excised from the polycyclic structure during ionization and fragmentation in the mass spectrometer. While we were not able to get solid experimental proof for this cleavage, such an excision resulting in the loss of a carbonyl group and yielding an [M-28]^+^ peak has been observed for other indole derivatives that structurally resemble ScyM (Rodríguez et al., 1996).

While the MS profile confirmed the existence of ScyM in the crude material, the PDA and mass spectra meanwhile revealed several other minor peaks (see Supplementary Figures S6A,B) which were impurities from the chemical synthesis (possibly remaining precursors and/or byproducts of the synthesis). For example, the *m/z* 123 peak eluting at RT = 2.78 min was recognized to be remaining 4-hydroxybenzaldehyde (Scheme 1, compound **5**) that was used as starting material in ScyM’s synthesis. As a result, a further HPLC purification was performed using ~10 mg of crude ScyM dissolved in MeOH, and yielded 4.4 mg of pure material. This purified ScyM was assayed by LCMS and showed a much better purity profile with a clean *m/z* 275 peak plus fewer and less intense impurity peaks (Supplementary Figures S8A,B). While a few very minor impurity peaks remained in this purified sample of synthetic ScyM, it was considered of sufficient purity for further tyrosinase inhibition assays.

The NMR data for synthetic ScyM (**1**) provided additional validation of its structure (see Table 1 with numbering pattern indicated in Supplementary Figure S9). The solvent peaks of DMF-d_7_ (^1^H: 8.03, 2.92, 2.75; ^13^C: 163.15, 34.89, 29.76) were used as internal chemical shift references. Additionally, 2D NMR spectra including HSQC and HMBC were performed to unequivocally assign carbon and proton atoms (Supplementary Figure S10); all results corresponded well with previously reported data (Suyama, 2009). Together, the results from LCMS and NMR confirmed the identity and purity of synthetic ScyM (**1**), and thus allowed for further biochemical and bioassay studies.

### Tyrosinase Inhibitory Activity of ScyM (**1**) in Comparison With KA (**3**) and Other Known Inhibitors

**Figure 4:**
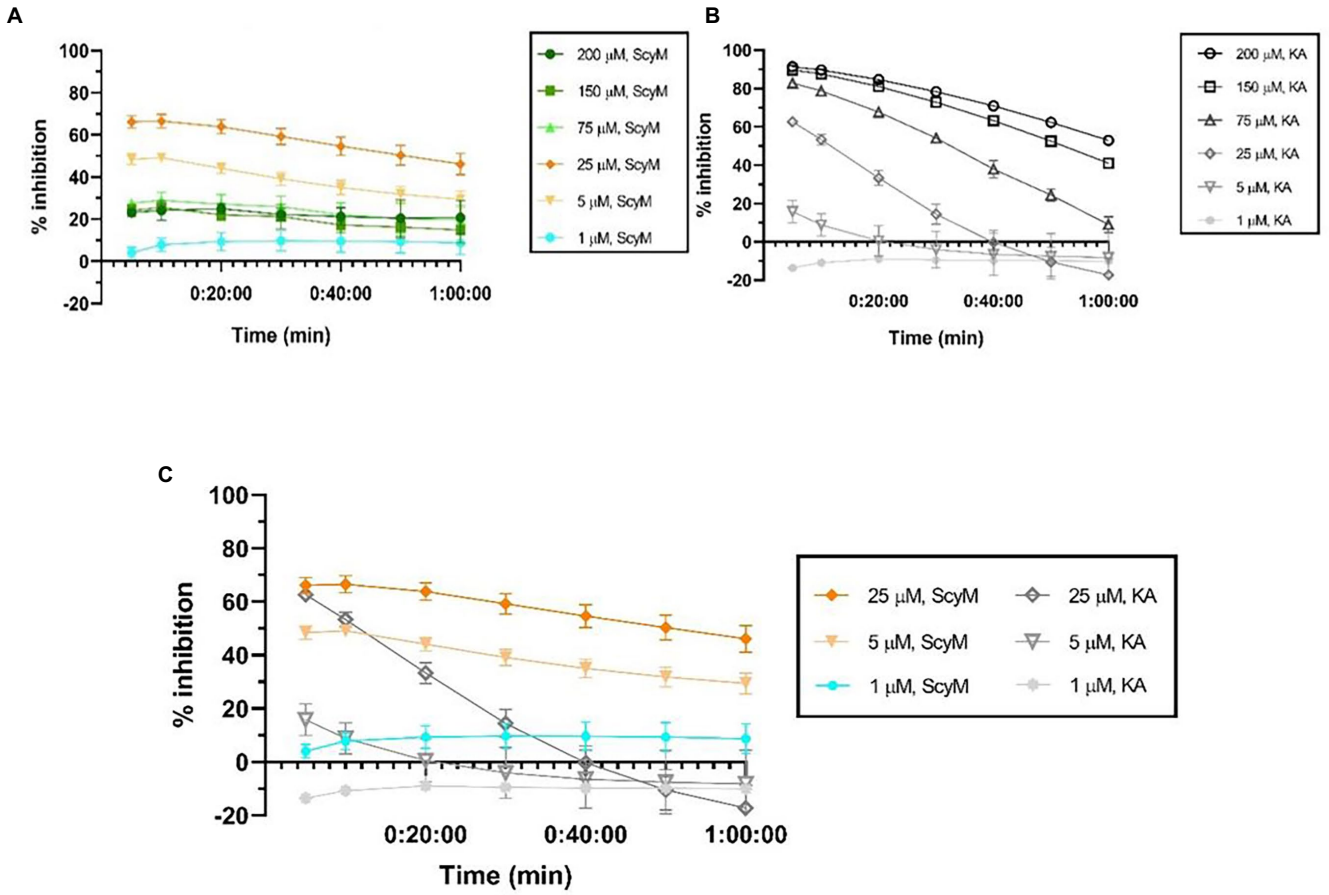
Dose-dependent percent inhibition against tyrosinase over time for: **(A)** scytonemin monomer (ScyM, **1**); **(B)** kojic acid (KA, **3**), the positive control compound; **(C)** ScyM and KA at 1, 5, and 25 μM concentrations at which ScyM completely dissolves in the 0.067 M sodium phosphate buffer and does not have solubility problems. Experiments were carried in triplicate and error bars represent standard deviation.

As a first evaluation of the inhibitory activity for the newly synthesized ScyM, a dose-dependent assay was performed in dynamic mode for both ScyM and the KA control. The final concentrations of the samples were set in μM, such that the inhibitory potencies could be easily compared. Newly synthesized but unpurified ScyM (e.g., material profiled in Supplementary Figure S6) was used for these initial confirmation assays. Figures 4A,B provide the results of the calculated time-dependent inhibition of these two compounds.

The inhibition of tyrosinase by KA (**3**) met our expectation in that the inhibition was more complete upon increasing concentrations (Figure 4B). However, while the percent inhibition for ScyM showed an increasing trend at lower concentrations (1 μM, 5 μM and 25 μM), once the concentration was increased to 75 μM or above, inhibition was unexpectedly decreased to approximately 25% and stayed unchanged despite increasing concentrations (Figure 4A). Upon repeated assay at higher concentrations of ScyM (e.g., 75, 150 and 200 μM), it was observed that there was precipitated material that accumulated at the bottom of the ScyM assay wells. This precipitation of ScyM was detected to occur when the buffer was added to the assay system, which is before the addition of tyrosinase enzyme and l-DOPA substrate. This suggested that ScyM might reach its saturation point in the assay system at a concentration less than 75 μM. ScyM’s poor solubility in water or buffer is in agreement with previous reports (Suyama, 2009). Consequently, we concluded that this solubility issue was responsible for the irregular inhibition trend described above. Undissolved ScyM significantly impedes the proper measurement by the plate reader, leading to an improper OD reading and hence a mistaken inhibition value. As a result, we conclude that the higher-concentration results for ScyM between 75 and 200 μM in Figure 4A are invalid and not reflective of the real inhibition properties of ScyM (**1**).

Therefore, removing the high ScyM concentration results and only considering those where ScyM was completely dissolved (Figure 4C), the tyrosinase inhibitory activity of ScyM was proportional to its concentration, just as for KA. Moreover, the inhibition by ScyM was always greater than KA when they were compared at the same concentration, indicating that the tyrosinase inhibitory potency of ScyM is greater than that of KA. Additionally, ScyM’s inhibitory effect against tyrosinase appeared to be maintained for a longer time period than KA in that its decrease in percent inhibition over time was less than that of KA (Figure 4). Consequently, these two attributes of ScyM’s inhibition suggested that ScyM might be a better tyrosinase inhibitor than commercially available KA. It is also important to note that the modest decrease in inhibition of tyrosinase by KA over time matched that of published results. This was previously interpreted as indicating that the KA-tyrosinase complex undergoes a “relatively slow reversible reaction” (Cabanes et al., 1994). Accordingly, ScyM might be an “even slower” reversible inhibitor compared to KA, as shown by its greater persistence of inhibition over time.

**Figure 5:**
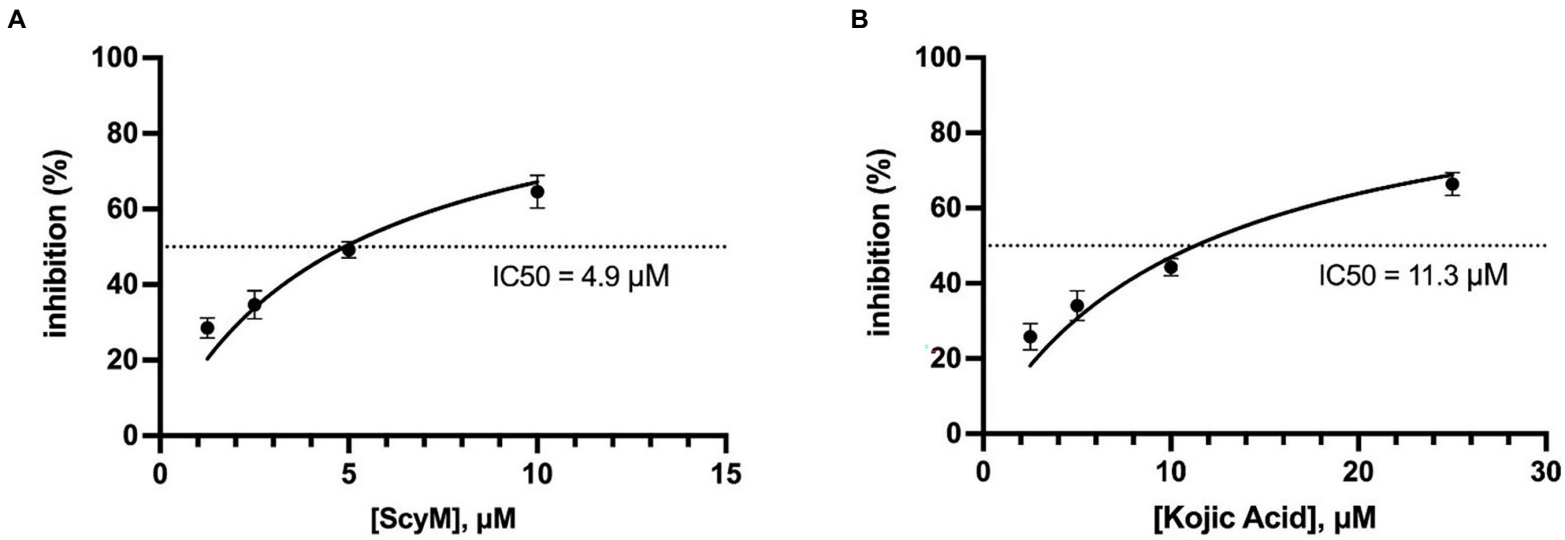
Calculated curves of tyrosinase inhibitory effect versus inhibitor concentration. **(A)** Scytonemin monomer (ScyM, **1**); **(B)** kojic acid (KA, **3**). The dotted lines represent the IC_50_ values, which were 4.9 μM for ScyM and 11.3 μM for KA, respectively. Experiments were carried in triplicate and error bars represent standard deviation.

Due to the solubility problems of ScyM in the assay buffer system used in these experiments, it was determined that all tyrosinase inhibitory assays with ScyM should subsequently be carried out in the range of 0–10 μM. At these concentrations, ScyM fully dissolves with no precipitate and shows strong and reproducible inhibitory activity. Therefore, to generate accurate IC_50_ values for ScyM (and KA), HPLC purified ScyM material was used at concentrations from 0 to 10 μM. As a result, ScyM was calculated to have a lower molar IC_50_ value compared to KA (4.9 vs. 11.3 μM, respectively; see Figure 5; Table 2), and supports our previous finding that ScyM has a stronger inhibitory effect against mushroom tyrosinase than the KA control. This value for KA of 11.3 μM was within the previously reported range of IC_50_ values, and hence helps to validate our overall findings and assay results (Neeley et al., 2009).

**Table 2:** Kinetic and inhibition constants of tyrosinase by ScyM and KA.

	Substrate	K_m_ (mM)	V_max_ (OD·min^−1^)	IC_50_ (μM)	Inhibition mode
ScyM	L-Dopa	0.24 ± 0.05	5.98 ± 0.52	4.9 ± 0.3	Mixed
KA	L-Dopa	0.51 ± 0.10	13.53 ± 1.48	11.3 ± 0.8	Mixed

Results were calculated as an average of triplicate measurements with SD.

Furthermore, the IC_50_ value of ScyM was also compared to other tyrosinase inhibitors reported in the literature (Table 3). To provide perspective on the mixed-type inhibitory potential of ScyM (determined by kinetic studies described below) as well as its marine origin, six other mixed-mode tyrosinase inhibitors (Table 3, compound 9–14) and six other marine-derived tyrosinase inhibitors (Table 3, compound 3–8), were chosen for comparison. Among the mixed-mode inhibitors, ScyM possesses the strongest tyrosinase inhibitory activity. And in comparing to other tyrosinase inhibitors of marine origin, such as the extracts of marine fungi (Li et al., 2005; Wu et al., 2013), ScyM equated favorably in terms of relative inhibitor potency. Furthermore, the inhibitory potency of ScyM was much stronger than oscillapeptin G (IC_50_ ≈ 100 μM post-conversion), which is the only other reported tyrosinase inhibitor from a cyanobacterial source (Sano and Kaya, 1996).

**Table 3:** Mushroom tyrosinase inhibitory activity of selected compounds from previous publications, in comparison with the positive control used in this experiment (KA) and the newly discovered inhibitor scytonemin monomer (ScyM).

	Compound	IC_50_ (μM)	Mechanism	References
1	Scytonemin monomer (ScyM, **1**)	4.90 ± 0.33	Mixed	N/A
2	Kojic acid (KA, **3**)	11.31 ± 0.81	Mixed	N/A
3	2,3,6-Tribromo-4,5-dihydroxybenzyl methyl alcohol (extract of *Symphyocladia latiuscula*, a marine red alga)	270.53 ± 2.04	Competitive	Paudel et al., 2019
4	Bis-(2,3,6-tribromo-4,5-dihydroxybenzyl methyl ether; extract of *Symphyocladia latiuscula*, a marine red alga)	110.91 ± 4.95	Competitive	Paudel et al., 2019
5	6-n-Pentyl-α-pyrone (extract of marine-derived fungus *Myrothecium* sp.)	0.8^*^	—^**^	Li et al., 2005
6	Myrothenone A (extract of marine-derived fungus *Myrothecium* sp.)	6.6^*^	—^**^	Li et al., 2005
7	1β,5α,6α,14-Tetraacetoxy-9α-benzoyloxy-7β H-eudesman-2β,11-diol (extract of marine-derived fungus *Pestalotiopsis* sp. Z233)	14.8^*^	—^**^	Wu et al., 2013
8	4α,5α-Diacetoxy-9α-benzoyloxy-7βH-eudesman-1β,2β,11, 14-tetraol (extract of marine-derived fungus *Pestalotiopsis* sp. Z233)	22.3^*^	—^**^	Wu et al., 2013
9	(*E*)-2-Acetyl-5-methoxyphenyl-3-(4-methoxyphenyl)acrylate	8.3^*^	Mixed	Sheng et al., 2018
10	(*E*)-2-Isopropyl-5-methylphenyl-3-(4-hydroxyphenyl)acrylate	10.6^*^	Mixed	Sheng et al., 2018
11	Baicalein	110^*^	Mixed	Guo et al., 2018
12	3-Phenylbenzoic acid (3-PBA)	36.3^*^	Mixed	Oyama et al., 2016
13	Terephthalic acid	26,600 ± 2,040	Mixed	Yin et al., 2011
14	Brazilein	21,210 ± 825	Mixed	Hridya et al., 2015

* Standard deviation not specified in the source paper.

** Inhibition mode not specified in the source paper.

It was also interesting that the inhibition by purified ScyM at 10 μM (Figure 5A) was about the same as that obtained from previous experiments using crude ScyM at 25 μM after the same 5 min of l-DOPA addition (see Figure 4C), both being approximately 60% inhibition. We interpret these results to indicate that highly purified ScyM provides stronger tyrosinase inhibitory activity than less pure samples, as would be expected.

### Synergistic Effects Between ScyM and KA

The potential synergism and antagonism of drug combinations has been extensively studied in pharmacology. However, in the scope of tyrosinase inhibitor studies, most research has focused only on the inhibitory properties of single compounds rather than mixtures. Therefore, there is very limited information on the synergistic effect of tyrosinase inhibitors (Yu Q. et al., 2019). For the present study, the widely used isobologram method was selected for this analysis (see Supplementary Figure S11 for concept and sample model).

An isobologram for ScyM and KA was generated with respect to tyrosinase inhibition, based on another round of dose-dependent assays. The IC_50_ values of ScyM and KA were again determined, with IC_50_ of ScyM = 4.7 μM and IC_50_ of KA = 10.8 μM, both being close to previous measurements (4.9 μM and 11.3 μM respectively). Next, different concentrations of ScyM and KA with a 1:2.5 ratio were mixed and tested, and the IC_50_ of the mixture was determined as 2.05 μM ScyM +5.12 μM KA. With these IC_50_ values, the isobologram for ScyM and KA was constructed (Supplementary Figure S12A). The calculated IC_50_ of the mixture is located below the additive line, suggesting that there is a modest level of synergistic inhibition of tyrosinase between ScyM and KA.

Due to limited capability of the traditional isobologram analysis to evaluate the intensity of drug interactions, a complementary method was employed to further evaluate the potential for synergism between ScyM (**1**) and KA (**3**). An interaction index (*γ*) has been used to help better understand and mathematically describe the strength of the interacting effect of drugs (Huang et al., 2019). Based on the derived equation,$\gamma =\overset{n}{\underset{i=2}{\sum }}\frac{d_{i}}{D_{i}}$, where $D_{i}$ represents the dose of an individual drug to achieve 50% efficacy when acting alone, and $d_{i}$ represents the dose of each drug in the compound mixture when the combined drug achieves 50% efficacy. Accordingly, when the interactions between drugs are synergistic, additive, and antagonistic, the corresponding values of γ are <1, = 1, and >1, respectively. Therefore, the smaller the index value, the stronger the synergistic effect. In our case here, the 50% efficacy is equivalent to 50% of tyrosinase inhibition or the IC_50_ values. As a result, the interaction index for ScyM and KA was calculated to be approximately 0.91, a value that is smaller than 1 and hence confirms the predicted synergistic effect between ScyM and KA by the traditional isobologram method. However, because this value is close to 1.0, it also indicates that there is only a very slight synergism, which is again consistent with the limited concavity of the isobologram (Supplementary Figure S12A).

### Kinetic Analysis of Mushroom Tyrosinase and ScyM

**Figure 6:**
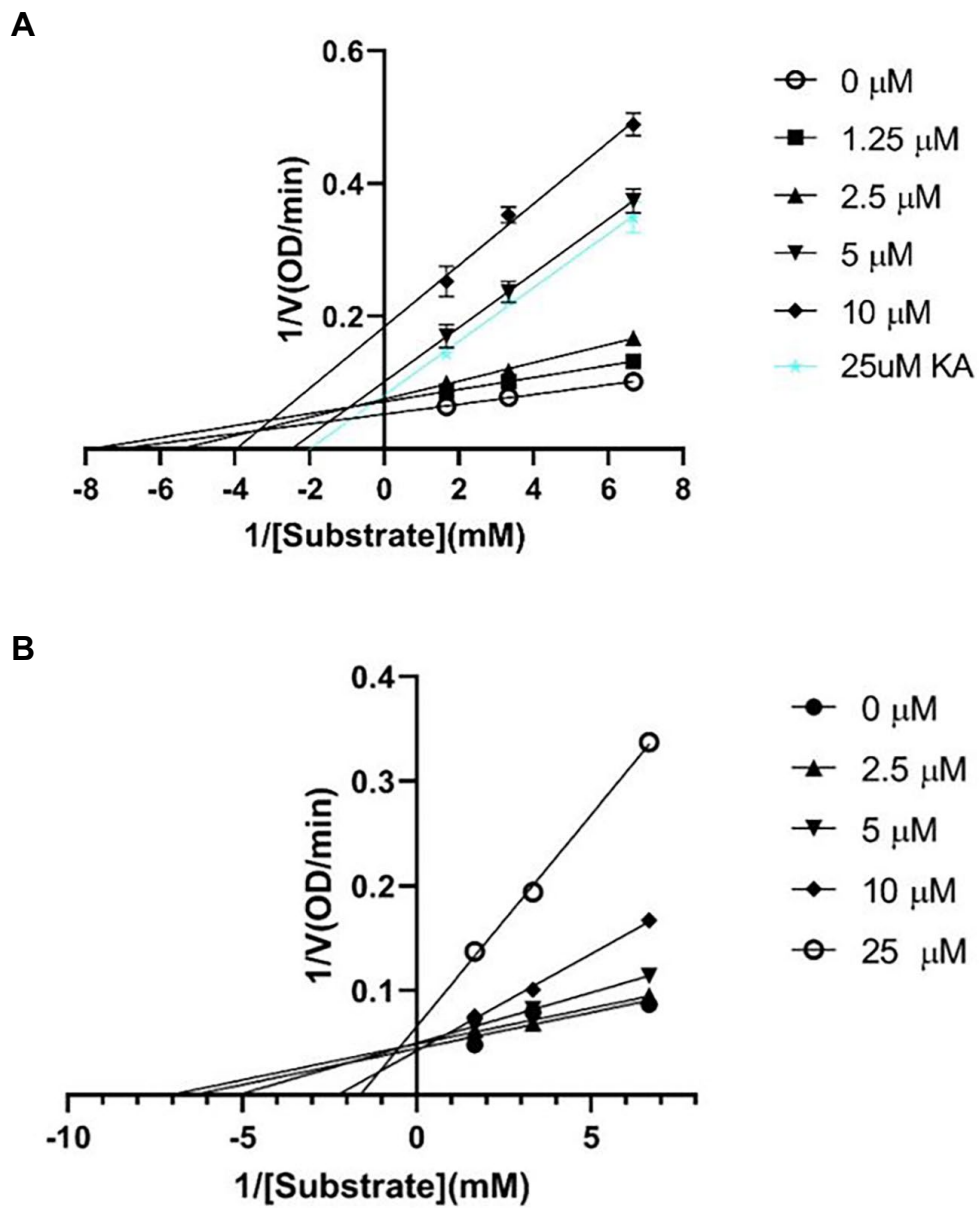
Lineweaver-Burk plots for inhibition against mushroom tyrosinase of **(A)** ScyM (**1**), and **(B)** KA (**3**). The blue line in **(A)** represents KA at 25 μM, which was used as the positive control in the assay. Experiments were carried in triplicate and error bars represent standard deviation.

A kinetic study was performed to better understand the tyrosinase inhibitory properties of ScyM. For this kinetic mode analysis, varying concentrations of substrate and inhibitor were used in the assays in order to generate Lineweaver–Burk plots. The substrate and inhibitor concentrations used in these experiments were similar to those used in previous research (Kim et al., 2020). The Lineweaver–Burk plot (Figures 6A,B), also known as the double reciprocal plot, is commonly used to determine the mechanism of how an inhibitor is inhibiting an enzyme. As its name suggests, the X-axis of the plot shows the reciprocal of substrate concentration ([S]), while the Y-axis shows the reciprocal of enzyme velocity ([V]). Two inhibition parameters, the Michaelis–Menten constant (*K*_m_) and maximal velocity (V_max_), are also visualized in this plot. The x-intercept represents −1/*K_m_* and the y-intercept represents 1/V_max_ (Fjellstedt and Schlesselman, 1977). The apparent *K*_m_ and V_max_ values were calculated as an average of triplicate measurements and are provided with standard deviations (see Table 2). The IC_50_ values representing inhibitor concentrations at which the enzyme activity is reduced by 50% are also included.

According to the Lineweaver-Burk plot (Figure 6B), with increasing concentrations of KA, the x-intercept moved rightward while y-intercept moved upward in the graph, demonstrating an increased *K_m_* but decreased V_max_; this is an indication of a mixed mode of inhibition, as suggested in a recent kinetic study elucidating the complex pattern of tyrosinase inhibition (Deri et al., 2016). Moreover, the plot itself was also consistent with a mixed inhibition mode, as both *K_m_* and V_max_ are affected at a non-proportional rate for this type of inhibition (Miesfeld and McEvoy, 2016). A similar pattern was also found for ScyM (Figure 6A) with a V_max_ smaller than KA (Table 2). Consequently, ScyM also inhibits mushroom tyrosinase in a mixture of competitive and uncompetitive modes, implying that it binds to both free enzyme and the enzyme-substrate complex (Zolghadri et al., 2019). The inhibition potency of ScyM was again shown to be stronger than that of KA, as indicated by its reduced enzyme velocity (V_max_). Interestingly, a mixed-mode of inhibition has been frequently observed in kinetic studies of mushroom tyrosinase inhibitors (Zolghadri et al., 2019). Many other compounds, such as terephthalic acid, brazilein and cinnamic acid ester derivatives, were also reported as mixed-mode mushroom tyrosinase inhibitors (Table 3). These other studies also used the Lineweaver–Burk plot analysis to make this conclusion on the mode of inhibition (Yin et al., 2011; Hridya et al., 2015; Sheng et al., 2018).

### Modeling the Interaction of Inhibitors and Substrates of Mushroom Tyrosinase *via* Molecular Docking

Molecular docking simulations can be used to predict the favored orientation of ligands toward target protein(s) to form stable complexes (Hassan et al., 2018). In this regard, *in silico* docking experiments are commonly used in enzyme studies as a complementary method to help understand the results of enzyme kinetic studies (Zolghadri et al., 2019). Using Molecular Operating Environment (MOE) software, different ligands including the compound of interest in this study ScyM (**1**), its *O*-methoxylated analog ScyM-OMe (**4**), the positive control KA (**3**) and the natural substrate of tyrosinase l-DOPA (see Figure 1 for structure), were separately introduced into the binding pocket of mushroom tyrosinase (PDB code: 2Y9X). This binding pocket is close to the binuclear copper-binding site that is coordinated by six conserved histidine residues (Supplementary Figure S13), as described in many of the previous research reports on this system (Ismaya et al., 2011; Lima et al., 2014; Karakaya et al., 2019). The outcomes from these docking experiments are shown in Table 4, including all ligand interactions that were present in the top five lowest energy results.

**Table 4:** Results from docking assays of various inhibitors and substrates with mushroom tyrosinase, including binding energies and common ligand interactions with the binding pocket residues.

	Structure	Best binding energy (Kcal/mol)	Second best binding energy (Kcal/mol)	H-bond interaction	Hydrophobic interaction	Metal/ion contact
l-DOPA	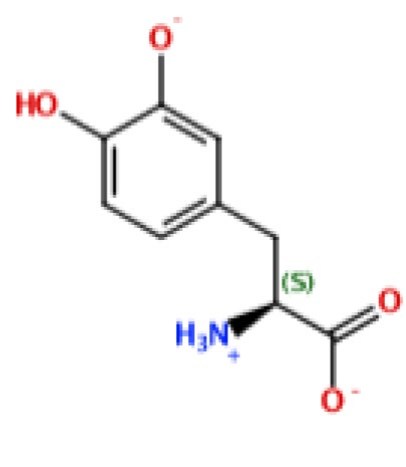	−7.20	−6.80	Val283 Ser282 Met280 Asn260	His263 Val283	Cu401 Glu256
Kojic acid	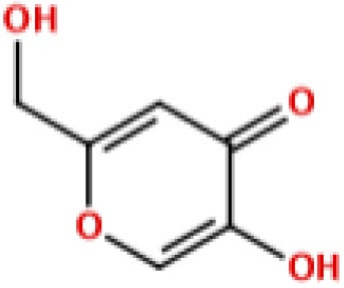	−5.51	−5.42	His85Met280	His263 Val283	Cu401
Deprotonated ScyM	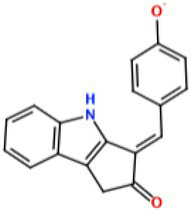	−7.16	−7.00	Val283 Ser282 Asn81	Val283 His85	Cu401
ScyM-OMe	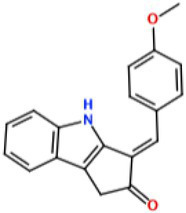	−5.74	−5.37	−	Val283	−

Note that Pi interactions include Pi-Pi interaction, cation-Pi interaction, and CH-Pi interaction.

**Figure 7:**
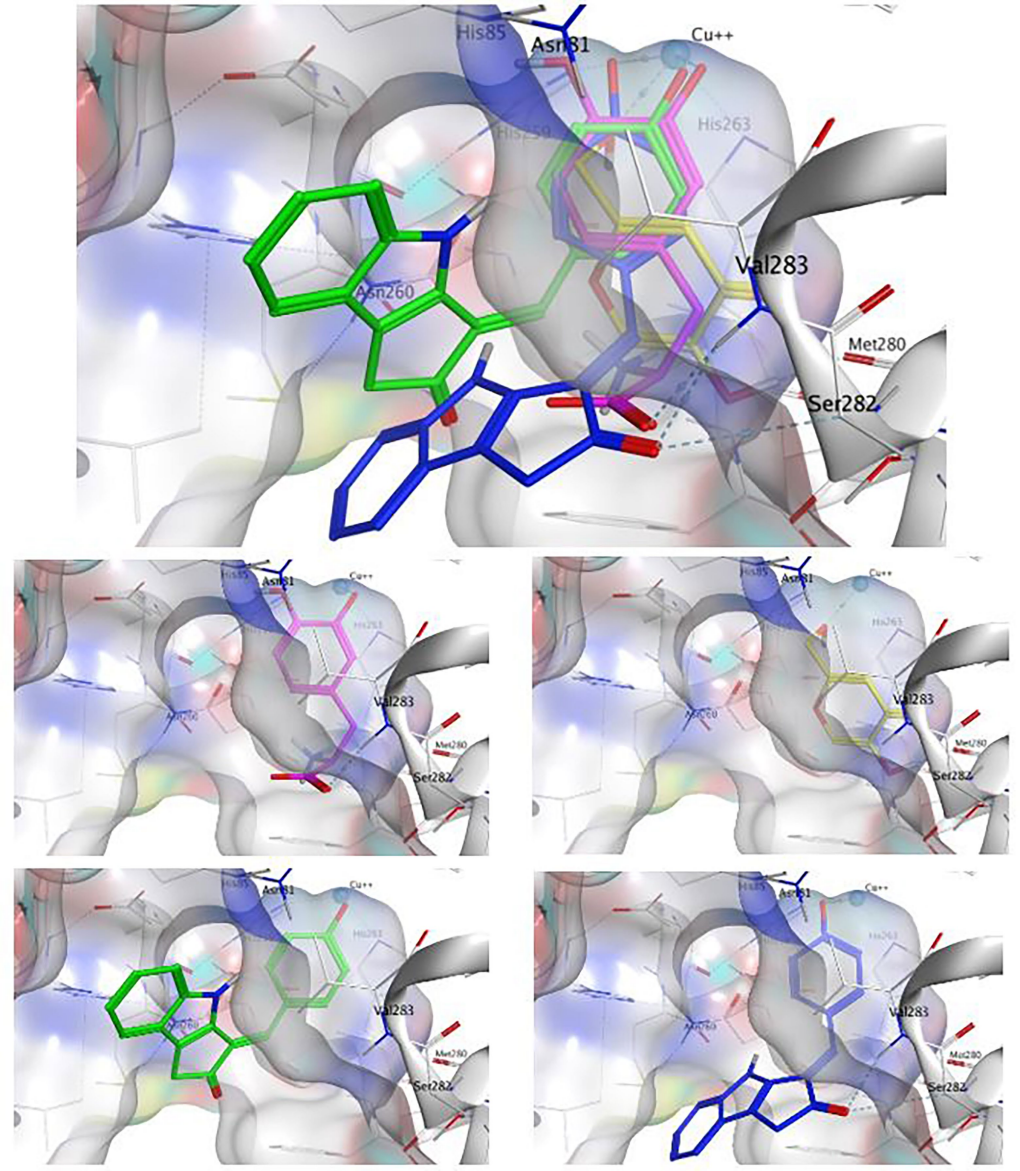
The binding conformation of l-DOPA (magenta), kojic acid (**3**, yellow), ScyM with best binding score (**1**, green) and ScyM with second-best binding score (**1**, blue). Sub-panels are provided for each individual compound to better show their interaction with nearby residues. The cyan sphere represents the copper ion in contact with ligands in substrate binding pocket. Blue colored dashed lines represent hydrogen bonds formed between ligand and corresponding tyrosinase residues. Dockings of various ligands were performed separately but assembled together in the composite figure for visualization of spatial overlaps.

After validation of docking accuracy using tropolone as described in the Methods section, the docking experiments started with the natural substrate l-DOPA. The structure of l-DOPA was extracted directly from the Protein Data Bank (PDB: 4P6S). According to the best predicted conformation of the L-DOPA substrate inside the binding pocket (Figure 7), the deprotonated phenol oxygen forms a metal contact with one of the copper ions. This agrees with reported catalytic functioning of tyrosinase, as the copper ions bind to the oxygen atom of the substrate and catalyze a redox reaction (Solano, 2018). Other important ligand interactions were also found: the carbonyl oxygen was found to form a hydrogen bond with Val283 and (or) Ser282 in the top 2 results, and Pi-Pi stacking and CH-Pi interactions were found for l-DOPA’s aromatic ring with His263 and Val 283, respectively. Additionally, some other interactions, such as H-bond formation between the positively charged nitrogen of l-DOPA with Met280 or Asn260, were indicated only for the binding orientations that were of somewhat lower binding energy (the lower three out of five).

The standard inhibitor, KA (**3**), was found to form a metal contact with the copper ion and to form a hydrogen bond with Met280 (Figure 7). Besides, the model also suggested Pi interactions with His263 and Val283. These results for KA were consistent with experimental data from previous studies, especially those for the interactions with the Cu^2+^ ion and Met280 (Kim et al., 2018; Koirala et al., 2018), and provided confidence in the validity of our docking experiments.

Next, the binding conformation ScyM (**1**) in mushroom tyrosinase was modeled, and the binding site was found to be close to that of the natural substrate l-DOPA. ScyM was also predicted to form a metal contact with the copper atom at the catalytic site (Figure 7, green and blue), and the phenol moiety of ScyM was found to extensively overlap that of the catechol of l-DOPA (Figure 7, green, blue, and magenta). These interactions could contribute to the competitive side of the mixed-type inhibition of ScyM, as ScyM would be predicted to strongly compete with the l-DOPA substrate for the binding site and consequently hamper catalysis. Further, this might explain why ScyM shows stronger inhibition compared to KA as this latter compound binds further away from the l-DOPA binding site (Figure 7, yellow and magenta). Another possibility is that, compared to ScyM, the lower binding energy of KA might constrain its ability to stabilize within the binding pocket and thus less efficiently inhibit substrate binding and catalysis. This analysis is in agreement with the intermediate binding site proposed in previous work where KA was found able to bind at the entrance to the active site of tyrosinase (Deri et al., 2016).

Several other ligand interactions were observed among the top results from docking of ScyM to mushroom tyrosinase, including an H-bond between ScyM and Val283 & Ser282, and CH-Pi interactions with Val283. There was also a predicted hydrogen bond formed with Asn81 in poses that were of lower binding energy. Overall, a majority of these interactions between ScyM and tyrosinase were found to be similar to those found with the native substrate l-DOPA, which further supported the competitive inhibition aspect of ScyM’s mixed-type inhibition. In addition, some of these interactions, such as the H-bonds with Ser282 and Asn81, as well as CH-Pi contact with Val283, matched with the docking results of other recently designed tyrosinase inhibitors (Karakaya et al., 2019). The contact with Cu^2+^ was similar to that reported for many previously found tyrosinase inhibitors, providing supporting evidence that copper chelation contributes to inhibition of the enzyme (Yin et al., 2011; Şöhretoğlu et al., 2018). This similarity in findings compared with preceding work increases confidence in the validity of our findings, and also enhances the possibility that part of ScyM’s inhibition of tyrosinase is mediated through metal chelation.

**Figure 8:**
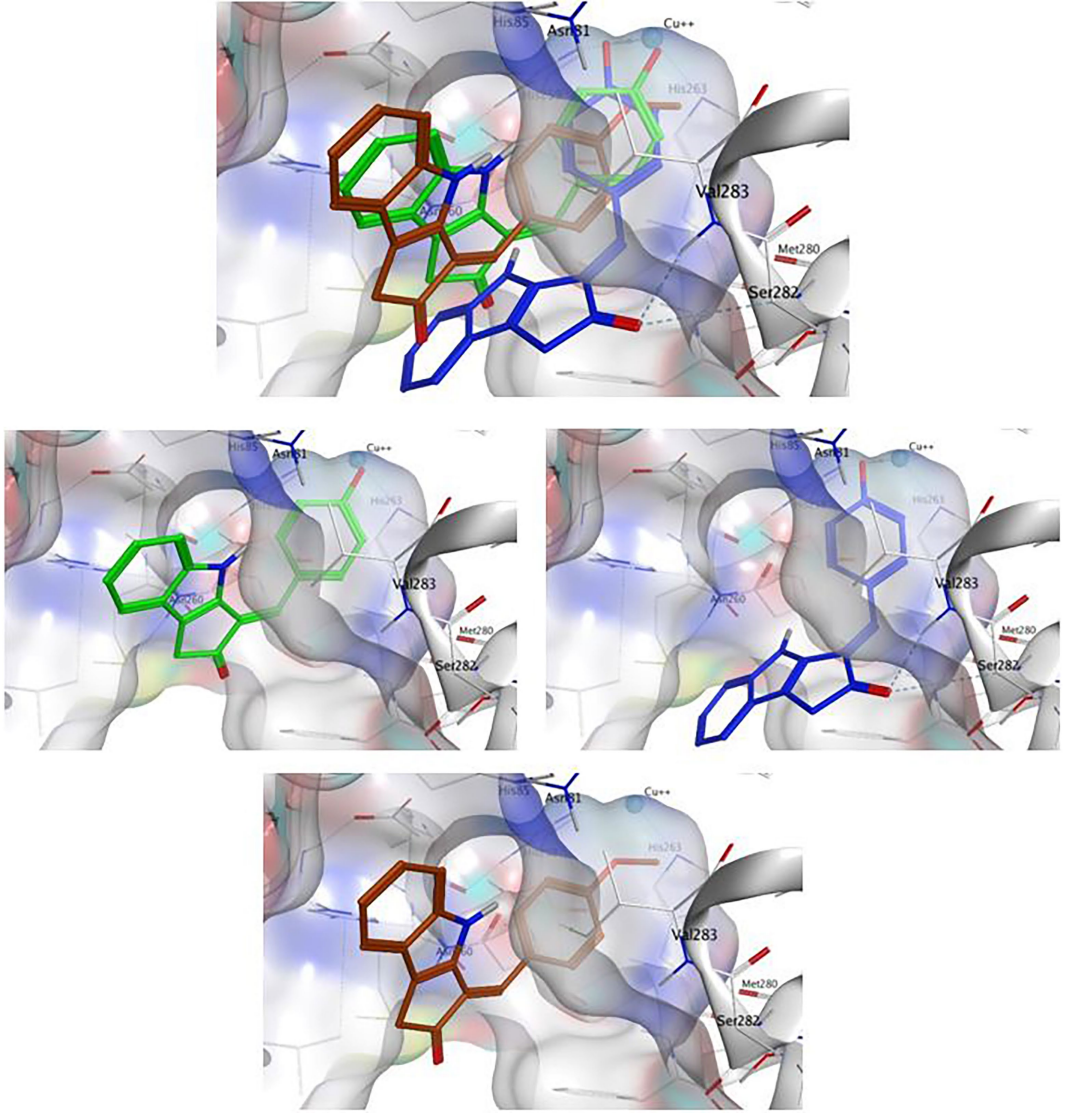
The binding conformation of ScyM with best binding score (**1**, green), ScyM with second-best binding score (**1**, blue) and ScyM-OMe (**4**, brown). Sub-panels were provided for each individual compound to better show their interaction with nearby residues. The cyan sphere represents the copper ion in contact with ligands in the binding pocket. Blue colored dashed lines represent hydrogen bonds between ligand and corresponding tyrosinase residues. Dockings of various ligands were performed separately but placed together in the same plot in the composite figure for visualization of spatial overlaps. The methoxylated monomer ScyM-OMe was no longer closely associated with the catalytic copper-binding site.

The methoxy analog ScyM-OMe was also subjected to a series of docking experiments. As shown in Figure 8, replacement of the phenol hydroxy group of ScyM by the more stable methoxy group eliminated association with the copper ion, and the phenyl ring was thus located further away from the catalytic site compared to pose 1 and pose 2 of ScyM. No interaction for ScyM-OMe was observed with tyrosinase except Pi-Sigma contact with the Val283 residue. Moreover, the binding energy decreased dramatically for ScyM-OMe, from above −7 Kcal/mol to only −5.74 Kcal/mol (Table 4). These findings corresponded with our experimental data from tyrosinase inhibitory assays subsequently performed with ScyM-OMe. At a concentration of 35 μM, there was only about 1% inhibition of tyrosinase activity, indicating very little inhibitory activity remaining after this structural modification. Therefore, from a combination of *in silico* docking and *in vitro* assays, it appears that the phenol moiety is largely responsible and necessary for the mixed-type tyrosinase inhibitory activity of ScyM. This conclusion agrees quite well with the literature which indicates that there is a strong correlation between potent tyrosinase inhibitory effects and compounds with hydroxy groups (Lima et al., 2014).

Lastly, besides the competitive aspect of inhibition described above, we hypothesize that the uncompetitive aspect of ScyM’s mixed-type inhibition might be the result of its also serving as substrate for the mushroom tyrosinase. Tyrosinase has both monophenolase (l-tyrosine as natural substrate) and diphenolase (l-DOPA as natural substrate) activities. Considering the similarity of the phenol head of ScyM which is shared by l-tyrosine (see Figure 1 for structure), it seems reasonable that tyrosinase may also be capable to catalyze hydroxylation of ScyM to form a diphenol, paralleling the conversion of l-tyrosine to l-DOPA.

### Inhibition of Tyrosinase by Scytonemin vs. Scytonemin Monomer

**Figure 9:**
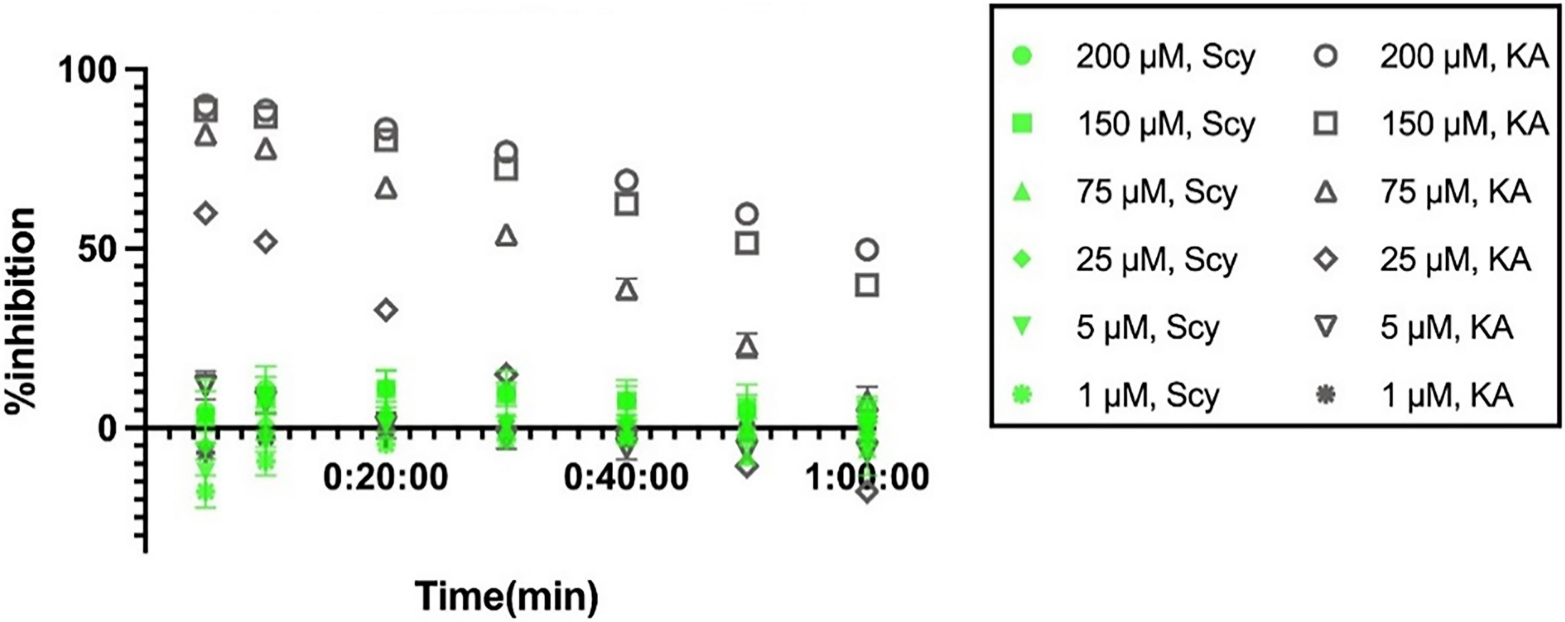
Dose-dependent percent inhibition of mushroom tyrosinase by scytonemin (**2**) and kojic acid (**3**) over time. Shown in green are the results of scytonemin and in gray are the results of the positive control kojic acid (KA). Experiments were carried in triplicate and error bars represent standard deviation.

The tyrosinase inhibitory properties that we found for ScyM led us to wonder if similar properties would be present for its dimeric structure, the cyanobacterial sunscreen pigment scytonemin (**2**). Therefore, a dose-dependent assay was also performed for scytonemin. Curiously, the results revealed that at all concentrations tested, from 1 up to 200 μM, there was almost no inhibition by scytonemin (Figure 9). These results were considered valid as the positive and negative controls functioned properly, showing percent inhibitions similar to previous assays (Figures 4B, 9). Therefore, we conclude that scytonemin is not a tyrosinase inhibitor. This might be because the dimeric scytonemin (**2**) molecule is too large to enter the catalytic site of tyrosinase and thus can no longer participate in ligand interactions with surrounding residues as does ScyM (**1**).

In order to further explore this hypothesis, an MOE docking experiment with scytonemin was performed (Supplementary Figure S14). The scytonemin molecule was inserted into the binding pocket of mushroom tyrosinase and evaluated for best poses. Unexpectedly, the docking result showed that deprotonated scytonemin could achieve a similar positioning inside the enzyme pocket to that of ScyM, and could make a similar metal contact with the Cu^2+^ just like l-DOPA, ScyM and the other substrates discussed above. The top two poses of scytonemin gave binding energy of −8.23 and −8.16 Kcal/mol respectively, indicating an even greater binding of scytonemin to tyrosinase than ScyM. This result suggests that scytonemin might also retain robust binding to mushroom tyrosinase, if its phenol head could be secured inside the binding pocket. However, it is most likely that this better binding score is partially attributed to the portion of its larger structure that is exposed outside of the pocket, and might hence be artificially higher and not reflective of its real binding affinity to the ligand binding site. In support of this hypothesis, scytonemin (**1**) is predicted to interact with Phe264 and Val248 residues of tyrosinase, both of which are quite distant from the catalytic copper-binding pocket and the conserved histidine residues (Supplementary Figure S14).

In addition to the possibility that *in silico* poses are over idealized and scytonemin is simply too large to have its phenol moiety properly bind in the substrate pocket *in vitro*, in its best five poses it was found to not form an H-bond with Val283 and Ser282 (Supplementary Figure S14), unlike ScyM (Table 4; Figure 7). The lack of a strong non-covalent interaction and only Pi interactions mediating the positioning of scytonemin alone may result in a lack of stabilization of the molecule in the substrate binding site. This would further result in a lack of contact by scytonemin with the copper ion which is crucial for inhibitory activity.

### Cytotoxicity of ScyM to Human Cancer Cells

While displaying difference in the anti-tyrosinase activity, scytonemin (**2**) and ScyM (**1**) show similar activities in the cytotoxicity assay. Our cytotoxicity assay revealed ScyM to be non-cytotoxic, as no significant effect was observed with H460 human lung cancer cells at concentration as high as 254 μM. Scytonemin was also reported to be non-cytotoxic in several research reports (Malla and Sommer, 2014; Kang et al., 2020).

## Conclusion

From a screening campaign of marine algal and cyanobacterial extracts, fractions and pure compounds using a colorimetric assay, we discovered scytonemin monomer (ScyM, **1**) to possess excellent *in vitro* mushroom tyrosinase inhibitory activity. Its inhibitory activity is greater than that of the commercially available tyrosinase inhibitor standard, kojic acid (KA, **3**), as well as many other tyrosinase inhibitors reported in the literature. We also revealed the ScyM retains slowly reversible mixed-type inhibition against mushroom tyrosinase. It was also shown that there is a modest level of synergistic effect between ScyM and KA regarding mushroom tyrosinase inhibition. On the other hand, the *O*-methoxylated analog of ScyM (**4**) and its dimeric form the cyanobacterial sunscreen pigment scytonemin (**2**), both showed complete loss of inhibitory activity *in vitro*. Overall, we successfully identified ScyM as the second case ever of a novel tyrosinase inhibitory compound based on a marine cyanobacterial natural product. Together with the fact that it is non-cytotoxic, ScyM can be a promising candidate for applications such as a skin-whitening agent or an adjuvant therapy for melanoma treatment. We hope this work opens the possibility that future research on other cyanobacterial-derived natural products, as well as some mycosporine-like amino acids (MAAs), will reveal additional anti-tyrosinase compounds with promising pharmaceutical and cosmeceutical value.

## Data Availability Statement

The original contributions presented in the study are included in the article/Supplementary Material, further inquiries can be directed to the corresponding authors.

## Author Contributions

WG and YH conceptualized and designed this study. TS performed the chemical synthesis of the compound of interest. EG performed the cytotoxicity assay. YH performed all other experiments included in this work. YH, HK, EG, and WG contributed to the data analysis. YH, WG, and TS contributed to the writing and revising of this manuscript. All authors contributed to the article and approved the submitted version.

## Funding

We gratefully acknowledge that this work was supported by NIH GM107550 (WG) and NSF-CHE-2102225 (TS).

## Conflict of Interest

The authors declare that the research was conducted in the absence of any commercial or financial relationships that could be construed as a potential conflict of interest.

## Publisher’s Note

All claims expressed in this article are solely those of the authors and do not necessarily represent those of their affiliated organizations, or those of the publisher, the editors and the reviewers. Any product that may be evaluated in this article, or claim that may be made by its manufacturer, is not guaranteed or endorsed by the publisher.
