# Supplementary material for: Discovery of Novel Tyrosinase Inhibitors From Marine Cyanobacteria
Source: Front Microbiol. 2022 Jul 13;13:912621. doi: 10.3389/fmicb.2022.912621 (PMC9329053; doi:10.3389/fmicb.2022.912621)
Supplement: Supplementary file 1 [file Data_Sheet_1.docx]

**Supporting Information**

**Discovery of Novel Tyrosinase Inhibitors from Marine Cyanobacteria**

Yifan He^1^, Takashi L. Suyama^2^, Hyunwoo Kim^1^, Evgenia Glukhov^1^ and William H. Gerwick^1, 3^

**Table of Contents**

**Table S1.** Percent inhibition by kojic acid at various final concentrations against tyrosinase.

**Figure S1.** A more comprehensive illustration of melanogenesis and involvement of tyrosinase in the pathway.

**Figure S2.** Layout of the colorimetric assay for screening for tyrosinase inhibition using 96-well microplates.

**Figure S3.** A diagram showing the proposed biosynthesis pathway of scytonemin in *Nostoc punctiforme* ATCC 29133.

**Figure S4.** Tyrosinase inhibitory activity of KA in a dose-dependent manner after 20 min of l-DOPA substrate addition.

**Figure S5.** Temporal shift of OD readings for: (A) blank with tyrosinase enzyme, (B) blank without enzyme, (C) KA with enzyme, (D) KA without enzyme, (E) ScyM with enzyme, and (F) ScyM without enzyme.

**Figure S6.** LCMS spectra of the synthesized crude ScyM product in the positive ionization mode.

**Figure S7.** MS^2^ spectrum of ScyM peak in the positive mode electrospray ionization (+ESI) with potential fragmentation of [M - 28]

**Figure S8.** LCESI MS spectra of post-HPLC purified ScyM in the positive ionization mode.

**Figure S9.** Atom numbering scheme for ScyM.

**Figure S10.** HSQC and HMBC correlations for ScyM in DMF-d_7_.

**Figure S11.** Illustration of a typical isobologram-based drug interaction analysis.

**Figure S12.** Isobologram analysis of interactions of ScyM and KA in tyrosinase inhibition.

**Figure S13.** Visualization of Mushroom tyrosinase (*Agaricus bisporus*) catalytic site.

**Figure S14.** Predicted ligand interaction of scytonemin and mushroom tyrosinase based on spatial orientation with highest binding score from docking experiment.

**Table S1.** Percent inhibition by kojic acid (**3**) at various final concentrations against tyrosinase. Results were calculated using OD measurements taken after 20 minutes of l-DOPA substrate addition. Experiments were performed in triplicate, with percent inhibition being calculated respectively and averaged. Results are presented as Mean ± SD.

| Concentration, µg/ mL | % Inhibition |
| --- | --- |
| 20 | 82.2 ± 1.1 |
| 10 | 62.2 ± 2.4 |
| 5 | 49.4 ± 0.4 |
| 2.5 | 28.9 ± 1.7 |
| 1 | 10.3 ± 1.1 |

**Figure S1.** A more comprehensive illustration of melanogenesis and involvement of tyrosinase in the pathway. Eumelanin and pheomelanin are listed as final products for instances of melanin. TYR stands for Tyrosinase, TYP-1 and TYP-2 refers to two tyrosinase-related proteins. Figure recreated from Pillaiyar et al., 2017.


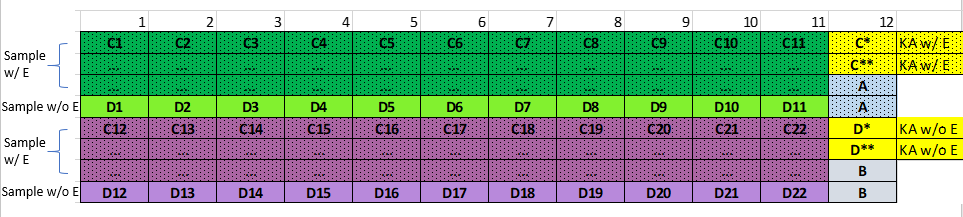


**Figure S2.** Layout of the colorimetric assay for screening for tyrosinase inhibition using 96-well microplates. Samples other than controls were run in triplicates. Four types of wells are shown above: A (Negative control with tyrosinase), B (Negative control without tyrosinase), C (Sample with tyrosinase, 3 wells as replicates), and D (Sample without tyrosinase = sample negative control), respectively. Wells C1-C22 and D1-D22 contained compounds to be tested, while C*, C** and D*, D** contained the known inhibitor kojic acid which served as a positive inhibitor control, run in duplicate.

**Figure S3.** A diagram showing the proposed biosynthesis pathway of scytonemin in Nostoc punctiforme ATCC 29133 and corresponding gene products involved in the biochemical steps. Arrows within gene cluster shows the direction of transcription. The main 18-gene cluster (Npun_R1276 to Npun_R1259) contains scyA, scyB, scyC, and tyrA, which encode the four key intermediate-forming enzymes, ScyA, ScyB, ScyC, and TyrA, respectively. Genes represented by the magenta arrows were responsible for the formation of pyruvic acid derivatives as precursors of scytonemin. An upstream two-component regulatory system (Npun_F1277 and Npun_F1278) regulates the expression of the main 18-gene scytonemin cluster. The set of 5 satellite genes (Npun_F5232 to Npun_F5236), referred to as ebo genes, encodes the Ebo complex. The Ebo complex exports the final precursor scytonemin monomer from the cytoplasm to the periplasm, where the monomers are dimerized to form the final product scytonemin with the help of mysterious enzyme(s) not yet determined. Figure recreated based on Pathak et al., 2020.


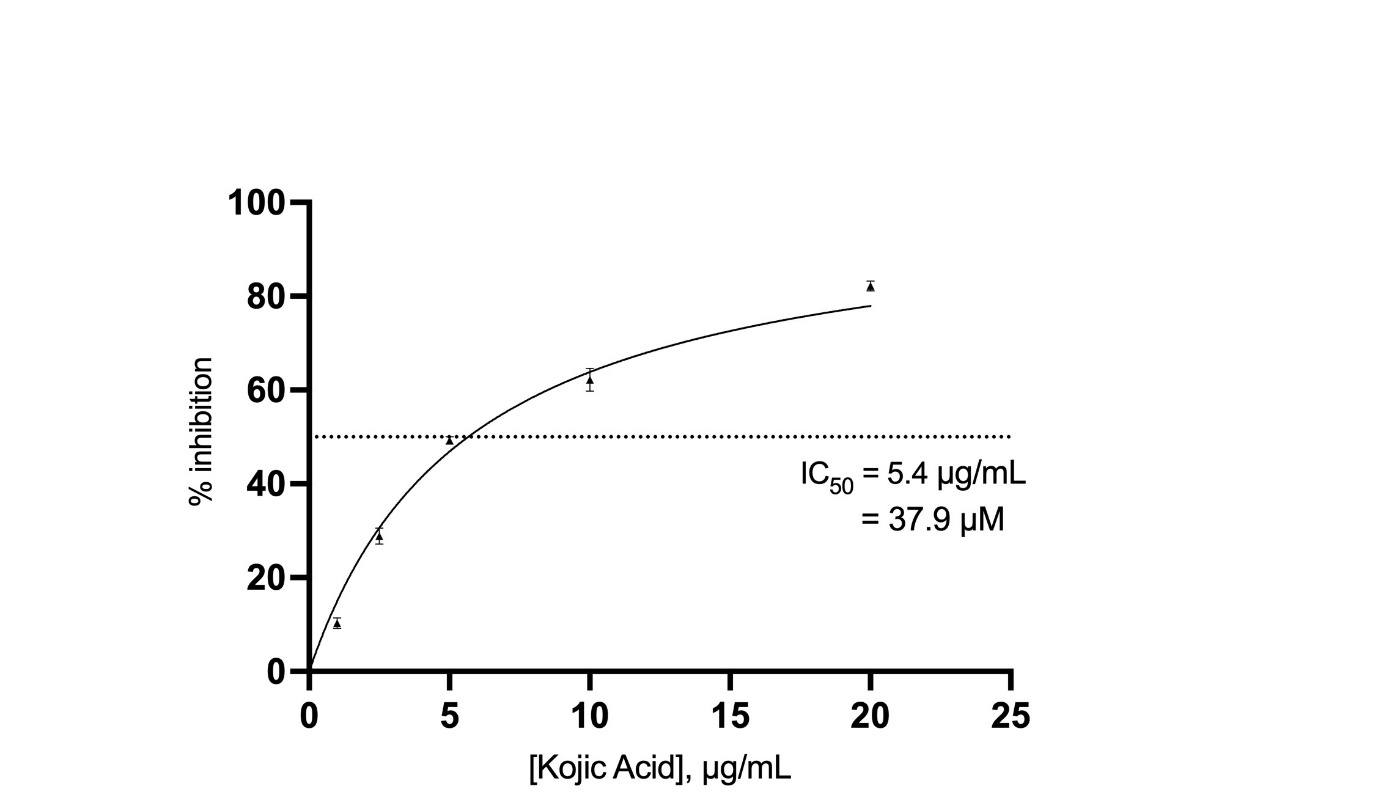


**Figure S4.** Tyrosinase inhibitory activity of KA (**3**) in a dose-dependent manner after 20 min of l-DOPA substrate addition. KA was tested in triplicate at 1 µg/mL, 2.5 µg/mL, 5 µg/mL, 10 µg/mL, and 20 µg/mL, and data were fit by a predicted logarithmic curve. The dotted line represents the IC_50_ value, 5.4 µg/mL, which is equivalent to 37. 9 µM. Error bars represent standard deviation.


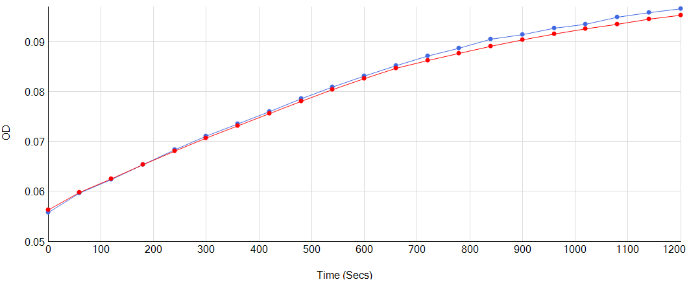

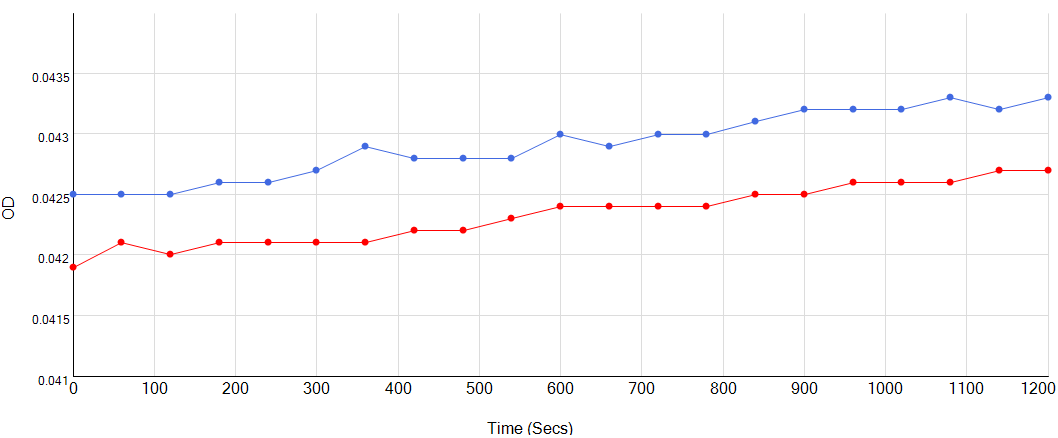


A B


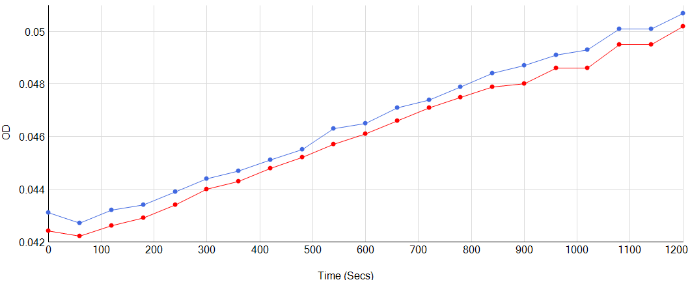

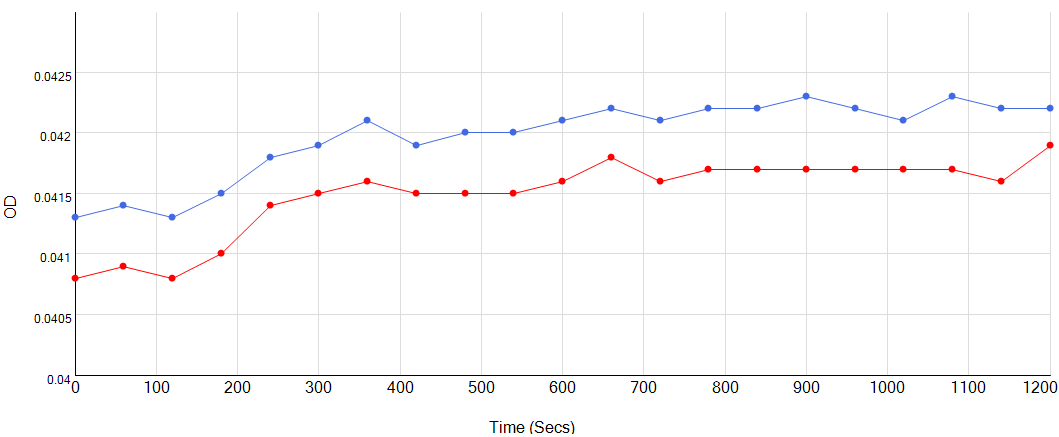


C D


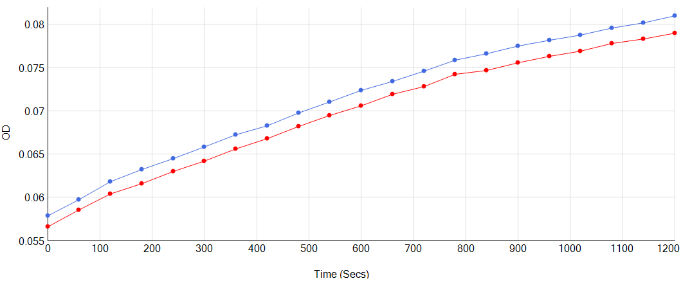

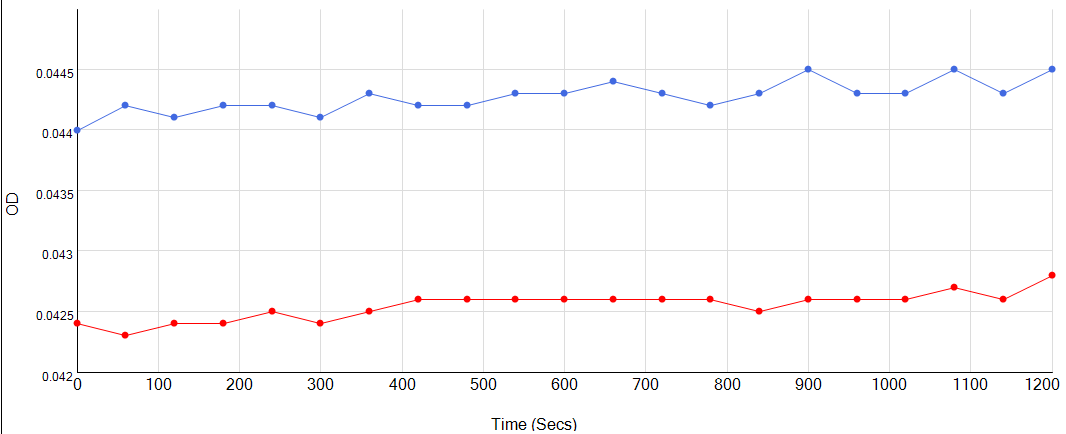


E F

**Figure S5.** Temporal shift of OD readings for: (A) blank with tyrosinase enzyme, (B) blank without enzyme, (C) KA with enzyme, (D) KA without enzyme, (E) ScyM with enzyme, and (F) ScyM without enzyme. Blue lines and red lines represent absorbance values at 475 and 490 nm, respectively.

Notes: Figures S4A and S4B illustrate the measured absorbance change within 20 minutes for the reagent mixture when no inhibitor was added. The increase in OD reading from ~0.055 to ~0.100 in S4A suggested that by the enzymatic addition of tyrosinase, there was a reasonable rate of conversion from L-DOPA to dopaquinone, which agreed with previously known knowledge that dopaquinone is more detectable than L-DOPA at the two measuring wavelengths. On the other hand, S4B shows a minor or no change in OD, which confirmed the negative control without enzyme addition. However, once the positive control KA or scytonemin monomer (ScyM) was introduced as inhibitors, the formation of dopaquinone was retarded, and the OD readings were therefore reduced to a level below 0.100, being ~0.050 and ~0.080, respectively (Figure S4C and S4E); meanwhile, the negative control experiments for these two inhibitors showed that without enzyme, the OD values were stable across the time period and were consistent with the values for the Blank control (Figure S4B, S4D and S4F). To conclude, in comparison with the controls, the OD changes observed with the inhibitors KA or ScyM were significant and thus due to a specific inhibition of the enzyme rather than being due to other random factors.

**A**

**B**

**Figure S6.** Liquid chromatography and mass spectrometry (LCESI MS) spectra of the synthesized crude ScyM product in the positive ionization mode. A) Total PDA detection with the major compound detected at the retention time (RT) of 9.54 min. B) Positive ion ESI MS monitoring of eluted peaks with the highest ScyM peak *m/z* 275 showing at RT = 9.76 min.


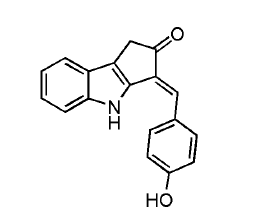


[M - 28] ^+^ *m/z* 275

**Figure S7.** MS^2^ spectrum of scytonemin monomer (**1**) peak in positive ion mode electrospray ionization (+ESI) showing a prominent [M - 28]^+^ fragment at m/z 247.

**A**

**B**

**Figure S8.** LCESI MS spectra of post-HPLC purified ScyM (**1**) in the positive ionization mode. A) Total PDA detection with major compound detected at the retention time (RT) of 9.53 min. B) Positive ion ESI MS monitoring of eluted peaks with the isolated ScyM peak m/z 275 showing at RT = 9.75 min. Both traces indicated a much better purity compared with the crude synthetic ScyM material.

**Figure S9.** Atom numbering scheme for scytonemin monomer (ScyM, **1**).


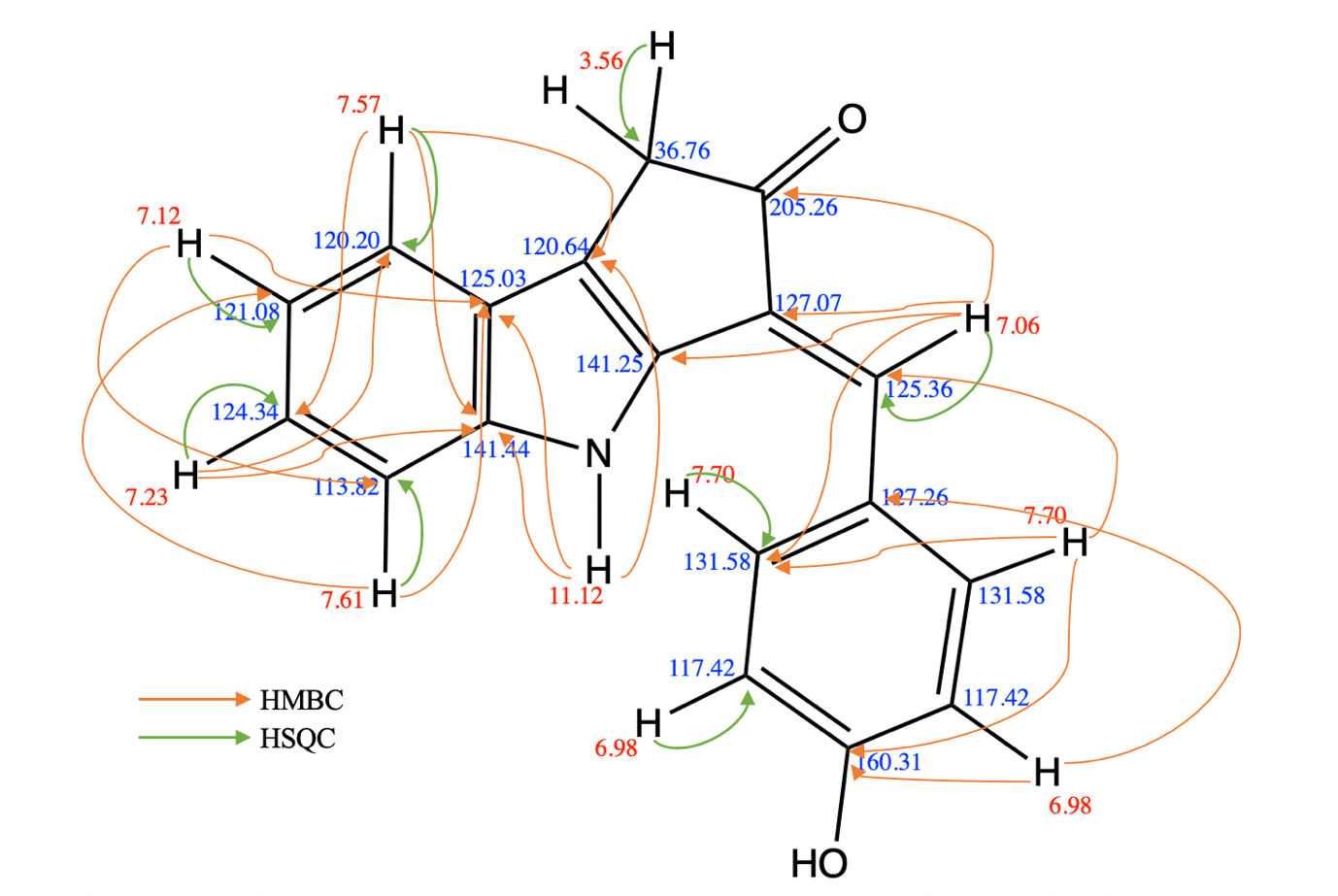


**Figure S10.** HSQC and HMBC correlations for scytonemin monomer (**1**) in DMF-d_7_. ^1^H NMR shifts are shown in red and ^13^C NMR shifts are shown in blue.


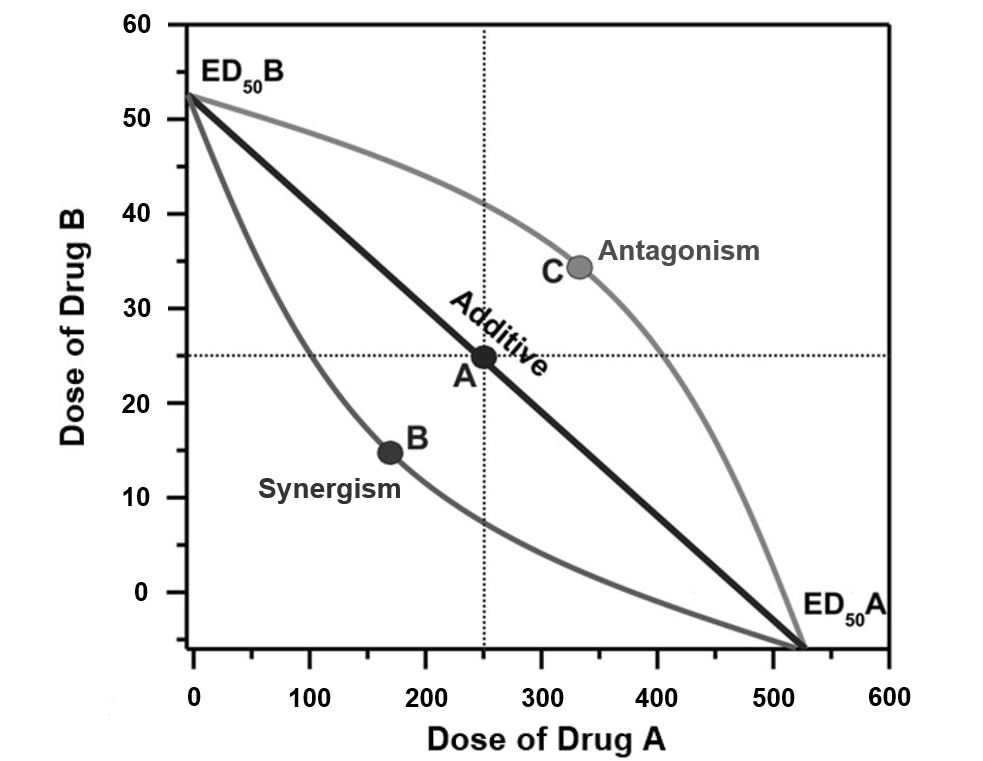


**Figure S11.** Illustration of a typical isobologram-based drug interaction analysis. The x- and y-axis represent the doses of drugs A and B, respectively. The solid black line connects doses that produce the same effect of examined response (commonly ED_50_). If the effects of drug A and drug B are independent of each other, the ED_50_ of mixture of A and B should theoretically fall on the straight additive line (point A). If the ED_50_ of mixture falls below the additive line instead (point B), meaning the same effect (50% efficacy) is achieved at lower doses of the two drugs, it indicates that there is an improved effect when the two drugs are combined, which is called synergism. Vice versa, if the ED_50_ of mixture falls above the additive line (point C), there is a reduced effect for the drug combination and the drugs hence exhibit antagonism. The dashed lines are shown to enhance the recognition of symmetry for additive isobole responses. Figure recreated from Chuang and Reddy, 2020.

(A)

(B) (C)

(D)

**Figure S12.** Isobologram analysis of interactions of ScyM and KA in tyrosinase inhibition. (A) IC_50_ Isobologram for ScyM & KA. The theoratical additive line connecting the IC_50_ values of ScyM and KA is shown in blue. And the concave grey line passing through the estimated IC_50_ value of mixture, indicates a potential synergistic effect between the two compounds. Sub-panels (B), (C) and (D) are predicted curves to help estimate the IC50 values of ScyM alone, KA alone, and mixture of ScyM and KA, respectively, to help the generation of isobologram in (A).


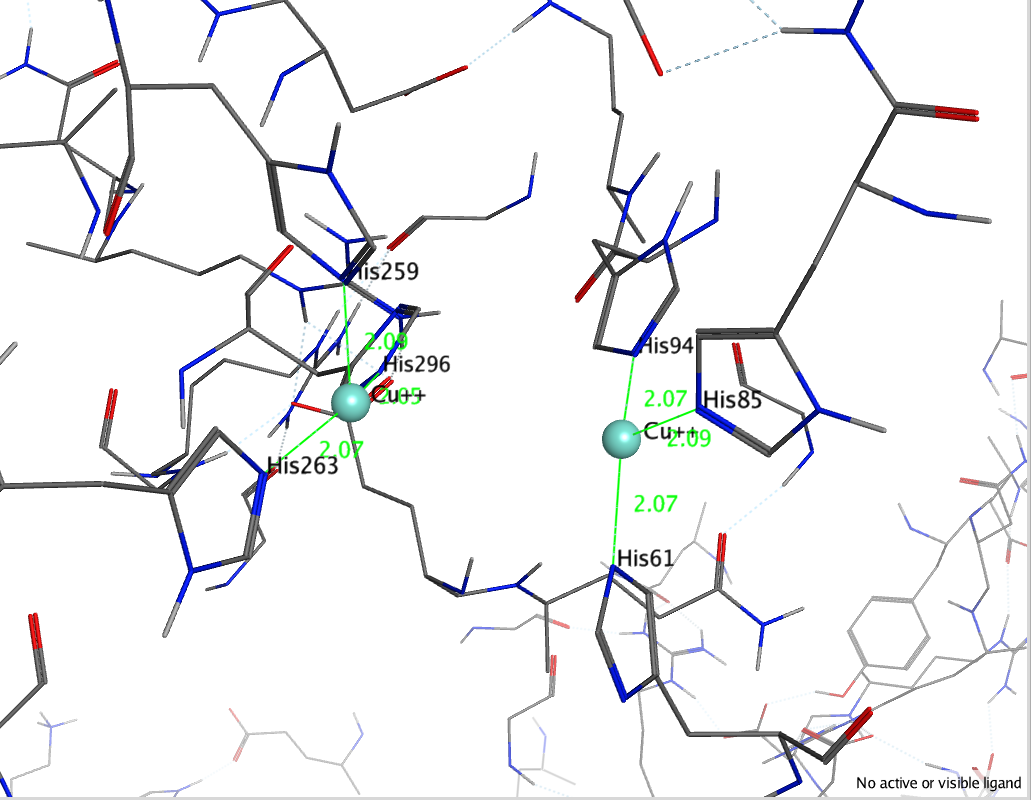


**Figure S13.** Visualization of Mushroom tyrosinase (Agaricus bisporus) catalytic site, with copper ions coordinated by His61, His85, His94, His259, His263 and His296 amino acid residues. All distances shown in green are in Å.


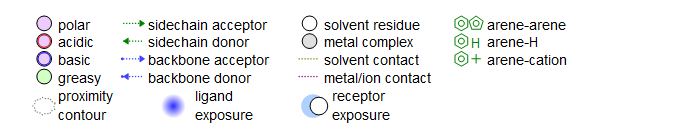


**Figure S14.** Predicted ligand interaction of scytonemin (**2**) and mushroom tyrosinase based on spatial orientation with highest binding score from docking experiment. Visualization generated by Molecular Operating Environment 2019 (MOE 2019.0102).

***Reference***

Chuang S-H, Reddy DS. (2020) Isobolographic analysis of antiseizure activity of the GABA type A receptor-modulating synthetic neurosteroids brexanolone and ganaxolone with tiagabine and midazolam. *J Pharmacol Exp Ther*. 372(3):285–298.

Pathak J, Pandey A, Maurya PK, Rajneesh R, Sinha RP, Singh SP. (2020) Cyanobacterial secondary metabolite scytonemin: A potential photoprotective and pharmaceutical compound. *Proc Natl Acad Sci India Sect B Biol Sci.* 90(3):467–481.

Pillaiyar T, Manickam M, Namasivayam V. (2017) Skin whitening agents: medicinal chemistry perspective of tyrosinase inhibitors. *J Enzyme Inhib Med Chem.* 32(1):403–425.
